# Evolutionary-Conserved Allosteric Properties of Three Neuronal Calcium Sensor Proteins

**DOI:** 10.3389/fnmol.2019.00050

**Published:** 2019-03-07

**Authors:** Valerio Marino, Daniele Dell'Orco

**Affiliations:** ^1^Section of Biological Chemistry, Department of Neurosciences, Biomedicine, and Movement Sciences, University of Verona, Verona, Italy; ^2^Department of Translational Research and New Technologies in Medicine and Surgery, University of Pisa, Pisa, Italy

**Keywords:** NCS1, recoverin, GCAP1, protein structure network, molecular dynamics, neuronal calcium sensors

## Abstract

Neuronal Calcium Sensors (NCS) are highly conserved proteins specifically expressed in neurons. Calcium (Ca^2+^)-binding to their EF-hand motifs results in a conformational change, which is crucial for the recognition of a specific target and the downstream biological process. Here we present a comprehensive analysis of the allosteric communication between Ca^2+^-binding sites and the target interfaces of three NCS, namely NCS1, recoverin (Rec), and GCAP1. In particular, Rec was investigated in different Ca^2+^-loading states and in complex with a peptide from the Rhodopsin Kinase (GRK1) while NCS1 was studied in a Ca^2+^-loaded state in complex with either the same GRK1 target or a peptide from the D_2_ Dopamine receptor. A Protein Structure Network (PSN) accounting for persistent non-covalent interactions between amino acids was built for each protein state based on exhaustive Molecular Dynamics simulations. Structural network analysis helped unveiling the role of key amino acids in allosteric mechanisms and their evolutionary conservation among homologous proteins. Results for NCS1 highlighted allosteric inter-domain interactions between Ca^2+^-binding motifs and residues involved in target recognition. Robust long range, allosteric protein-target interactions were found also in Rec, in particular originating from the EF3 motif. Interestingly, Tyr 86, involved the hydrophobic packing of the N-terminal domain, was found to be a key residue for both intra- and inter-molecular communication with EF3, regardless of the presence of target or Ca^2+^ ions. Finally, based on a comprehensive topological PSN analysis for Rec, NCS1, and GCAP1 and multiple sequence alignments with homolog proteins, we propose that an evolution-driven correlation may exist between the amino acids mediating the highest number of persistent interactions (high-degree hubs) and their conservation. Such conservation is apparently fundamental for the specific structural dynamics required in signaling events.

## Introduction

Calcium (Ca^2+^) is a universal second messenger whose changes in concentration contribute to the regulation of a variety of biological processes ranging from muscle contraction (Ebashi and Endo, 1968) to signal transduction (Berridge et al., 2003) and neuronal signaling (Augustine et al., 2003). The biological importance of Ca^2+^ ion requires a family of proteins called Ca^2+^-sensor proteins, which are able to change conformation in response to variations in intracellular Ca^2+^ concentration. Calmodulin is a ubiquitous Ca^2+^-sensor protein (Vetter and Leclerc, 2003) whose structural plasticity allows the regulation of a plethora of biological targets (Ikura and Ames, 2006). Neuronal Calcium Sensor proteins (NCS) though, are a tissue-specific and highly specialized class of proteins able to regulate a limited number of targets (Burgoyne, 2007; Burgoyne and Haynes, 2015) involved in a large array of neuronal transmission processes. Some sensors like Calcium and Integrin Binding Protein 2 (CIB2), for instance, are involved in hearing and vision (Riazuddin et al., 2012; Patel et al., 2015; Michel et al., 2017; Vallone et al., 2018), Recoverin (Rec) and Guanylate Cyclase Activating Proteins (GCAPs) are fundamental players of the phototransduction cascade (Koch and Dell'orco, 2013, 2015) and Neuronal Calcium Sensor 1 (NCS1) regulates sight (Baksheeva et al., 2015), synaptic plasticity (Saab et al., 2009), and neuronal differentiation (Dason et al., 2012). Moreover, NCS proteins may have only one predominant biological target, as is as case for Rec and GCAP1, or even regulate the same effectors, as is the case for the regulation of Rhodopsin Kinase (GRK1) by both Rec and NCS1.

A number of high resolution three-dimensional structures are available for some NCS proteins in their isolated states or in complex with peptides from their native targets (Ames and Lim, 2012). While being fundamental for understanding the mechanisms regulated by NCS proteins at atomistic resolution, static structures offer just a frozen picture of the indeed highly dynamic nature of such proteins and of the conformational changes that constitute the essence of their function. If a rigorous and consistent sampling of the conformational space is achieved, Molecular Dynamics (MD) simulations can complement structural information with time evolution to synergistically investigate even small conformational changes (Marino et al., 2015b) in NCS proteins (Marino and Dell'orco, 2016). Non-covalent interactions are continuously formed and broken in protein dynamic processes, however the most persistent ones are the physical forces responsible for the assembly of native protein structures. The time evolution of such persistent interactions monitored by MD simulations defines Protein Structure Network (PSN) rearrangements, which are a direct consequence of conformational variations in the proteins. PSN analysis can help identify allosteric properties (Schueler-Furman and Wodak, 2016; Greener and Sternberg, 2018) as well as residues of crucial importance in NCS proteins such as GCAP1 (Marino and Dell'orco, 2016).

Here we present a thorough analysis of PSN derived by MD simulations of Rec and NCS1 to assess potential allosteric mechanisms of intra- and inter-molecular information transfer that are related to specific signaling states. Moreover, we investigate the communication routes from Ca^2+^-binding sites to target interface, which allows us to identify residues that play an important role in establishing state-specific topology and in target communication. Finally, by analyzing residue conservation in homologs and among NCS1, Rec, and GCAP1, we infer evolutionary conserved properties regarding target interfaces and topological features of the PSN.

## Methods

### Structural Modeling of Rec and NCS1

The starting structures for MD simulations were either specific frames of deposited PDB files or partially modeled to avoid structural gaps. The structures with the highest resolution and sequence coverage available were chosen for both Rec and NCS1, resulting in the following models: Ca^2+^-free Rec (“tense Recoverin” or Rec-T, Tanaka et al., 1995), Rec with one Ca^2+^ bound to EF3 (“intermediate state Recoverin” or Rec-I, Ames et al., 2002), Ca^2+^-loaded Rec (“relaxed Recoverin” or Rec-R, Ames et al., 1997), Ca^2+^-loaded Rec bound to GRK1 peptide (Rec-GRK1, Ames et al., 2006; Zernii et al., 2011; Ames and Lim, 2012), Ca^2+^-loaded uncomplexed NCS1 (“isolated NCS1” or NCS1-iso), Ca^2+^-loaded NCS1 bound to D_2_ Dopamine receptor peptides (NCS1-D_2_R) and Ca^2+^-loaded NCS1 bound to Rhodopsin Kinase peptide [NCS1-GRK1, (Bourne et al., 2001; Pandalaneni et al., 2015)]. Details for molecular modeling are provided in Supplementary Methods.

### Molecular Dynamics Simulations

MD simulations of Rec and NCS1 states were performed using GROMACS 2016.1 simulation package (Abraham et al., 2015) and CHARMM36m (Huang et al., 2017) all-atom force field, where parameters for N-myristoylated Gly were generated manually (available upon request). Simulations were performed in a dodecahedral box with Periodic Boundary Conditions applied, where proteins were located at 12 Å distance from box boundaries. Solvent was modeled as TIP3P water, system was neutralized first with 1 mM MgCl_2_, then after addition of 150 mM KCl. The size for each simulated system was the following: 33,837 atoms for Rec-T, 38,878 atoms for Rec-I, 56,010 atoms for Rec-R, 86,456 atoms for Rec-GRK1, 46,809 atoms for NCS1-iso, 42,882 atoms for NCS1- D_2_R and 50,584 atoms for NCS1-GRK1.

All structures underwent substantially the same pre-production steps as previous studies concerning GCAP1 (Marino et al., 2015b), briefly consisting of steepest descent (*F* = 1,000 kJ/mol^*^nm) and conjugate gradients (*F* = 500 kJ/mol^*^nm) energy minimization, then equilibration at 310 K for 2 ns backbone-constrained and 2 ns unrestricted MD simulations in NVT ensemble. After equilibration, 200 ns unrestrained MD simulations were performed in NPT ensemble at 310 K and 1 atm for each system. Equilibration and production MD simulations were independently replicated 5 times by changing the random seed for initial velocity generation as previously described (Marino and Dell'orco, 2016), to achieve exhaustive sampling of the conformational space.

### Principal Component Analysis and Linear Discriminant Analysis

Collective protein motions were identified using Principal Component Analysis (PCA) after diagonalization of the Cα covariance matrix (Amadei et al., 1999) calculated on single 200 ns replicas and concatenated 1 μs trajectories for each protein form, after superimposition to the final structure of the equilibration phase of replica one. Eigenvalues and eigenvectors extracted from the diagonalized matrix represent amplitude and direction of collective motions (Principal Components, or PC) and are ranked in decreasing order of eigenvalues, meaning that the first PC (PC1) accounts for the largest collective motion, PC2 for the second largest and so on.

The Essential Subspace (ES) of the first 20 PC, accounting for 78–93% of total motion of Rec and NCS1 states, was identified as in Marino and Dell'orco (2016) to compare the conformational space sampled by each replica and 1 μs trajectories using the Root Mean-Square Inner Product (RMSIP) index (Amadei et al., 1999). Details for cosine content (*c*_1_) (Hess, 2002) and RMSIP index calculations are provided in Supplementary Methods.

The sampling convergence of MD simulations was also assessed by performing Linear Discriminant Analysis (LDA, Martinez and Kak, 2001) on the projection of the frames of the 5 replicas on PC1 and PC2 calculated on the concatenated 1 μs trajectories. This supervised classification method decreases the number of features describing data and combines them to find a hyperplane that maximizes separation between means of projected classes and minimizes the variance within each projected class. In our case a two-dimensional feature space (PC1 and PC2) was classified using a single Linear Discriminant (LD1) and if a single conformation from a replica was classified as possibly belonging to a different replica, then the two replicas could be considered as consistent and therefore concatenable. Data presented in Figures S3, S4 was smoothed using Kernel Density Estimation smoothing (Rosenblatt, 1956; Parzen, 1962).

### Protein Structure Network Generation and Analysis

Dynamic information from concatenated 1 μs trajectories was converted into a PSN using PyInteraph software (Tiberti et al., 2014), with the same parameters as in Marino and Dell'orco, 2016, the only exception being the mass of atoms defined according to CHARMM36m force field (Huang et al., 2017). Briefly, non-bonded interaction between side chains (H-bonds, electrostatic and hydrophobic interactions) was computed as a percentage of frames where distance and angle constraints were satisfied. Interactions were filtered using the hydrophobic cluster size criterion (Vishveshwara et al., 2009), therefore the size of the biggest hydrophobic cluster was calculated at 0.1% persistence intervals, and the persistence threshold (*p*_*T*_) was computed as in Marino and Dell'orco (2016) and rounded at the lowest decimal value. For each case *p*_*T*_ was calculated and all interactions above the threshold were joined in the unweighted PSN graph representing the specific protein state.

The degree of connectivity was calculated for all residues in each PSN, amino acids with ≥ 4 connections were considered hubs and reported in Tables S1–S3. The Communication Robustness index (*CR*, Marino and Dell'orco, 2016) of two residues permits the evaluation of the structural information transfer between amino acids within a protein three-dimensional structure, and to highlight potential allosteric mechanisms. Details for CR calculation are provided in Supplementary Methods. CR index was therefore computed to measure the communication between Rec bidentate Ca^2+^-coordinating residues E85 and E121 and GRK1 interface residues (Table S4, Ames et al., 2006; Zernii et al., 2011). Analogously for NCS1, CR index was calculated between bidentate Ca^2+^-coordinating residues E84, E120, and E168 and with GRK1 or D_2_R interface residues (Table S4, Pandalaneni et al., 2015). For the sake of clarity, only interface residues with the 5 highest CR values for each presented state were reported in Figures S5, S6. Moreover, intramolecular communication was evaluated by analyzing CR calculated between the myristoyl group of Rec and GRK1-interface residues (Figure S7A) and among the previously mentioned Ca^2+^-coordinating residues of Rec and NCS1 (Figure S7B), to investigate intramolecular communications between EF-hand motifs.

Finally, also intermolecular communication was investigated by computing CR index between GRK1 peptide residues that were structurally solved (L6-I16) complexed with both Rec and NCS1 and their respective EF-hand representatives (Figure S8).

For visualization purposes, pathways connecting EF-hand representatives and GRK1 residues with the highest CR were chosen according to the highest cumulative Selective Betweenness (SB, Marino and Dell'orco, 2016). Details for SB calculation are provided in Supplementary Methods.

### Sequence Alignment of Homologous NCS

Protein sequences for bovine Rec (Uniprot: P21457), human NCS1 (Uniprot: P62166), human GCAP1 (Uniprot: P43080) were subjected to Multiple Sequence Alignment (MSA) with Clustal Omega (Sievers et al., 2011) to evaluate residue conservation among homologous NCS. Moreover, each protein sequence was also subjected to MSA with Clustal Omega with up to 250 Uniprot sequences having at least 50% sequence identity (s.i.) and 80% sequence coverage with respect to the longest sequence in the cluster. In detail, Rec was aligned with 122 sequences from Uniref50_P21457 and NCS1 was aligned with 250 sequences from Uniref50_P62166. Human GCAP1 was instead aligned with 101 sequences from Uniref50_P46065, referring to the bovine sequence, constituting the seed for the linked UniRef50 cluster (s.i. 94%).

Residue conservation in UniRef50 clusters shown in **Figure 6** and in Tables S1–S3, S5 was calculated as the ratio between the number of sequences where the residue of the seed sequence was conserved and the total number of sequences in the cluster. When a residue was a hub in different signaling states, even with a different degree, only one occurrence of the residue was considered for the calculation of the average of a given hub degree. Average values for each protein were mediated and subjected to linear regression after passing Shapiro-Wilk (Shapiro and Wilk, 1965) (*p* = 0.05) and constant variance (*p* = 0.05) tests, with *R*^2^ = 0.62.

Hub degree shown in Figure 5 represents the highest hub degree exhibited by a residue in one of the simulated states for each protein, data about GCAP1 are taken from Marino and Dell'orco (2016).

Pairwise sequence alignments were performed with Needleman-Wunsch (Needleman and Wunsch, 1970) algorithm.

## Results

### Consistent and Exhaustive Conformational Sampling of Rec and NCS1 Signaling States Achieved by Molecular Dynamics Simulations

Rec-T, Rec-I, Rec-R, and Rec-GRK1 were simulated to evaluate the network of persistent interactions and any potential difference in the inter- and intra-molecular allosteric properties. Such differences are ascribable to the physiological conformational transition of the sensor as a consequence of the decrease of intracellular [Ca^2+^] upon activation of the phototransduction cascade (Ames et al., 1996). On the other hand, NCS1-iso, NCS1-GRK1, and NCS1-D_2_R were simulated to evaluate differential communication of the same protein in the absence and in the presence of different targets. Furthermore, the presence of the same target, namely GRK1, interacting with both Rec and NCS1 allowed for a comparative analysis of the communication routes between Ca^2+^-binding sites and a common target.

For each protein state 5 × 200 ns replicas of MD simulations were run; individual replicas and 1 μs concatenated trajectories were subjected to PCA of Cα motions to assess consistency and exhaustiveness, as previously described (Marino and Dell'orco, 2016). Cosine content c_1_ of PC1 was the first necessary condition to achieve sampling convergence, as it was demonstrated that values close to 1 are indicative of insufficient sampling (Hess, 2000; Papaleo et al., 2009). The c_1_ for the concatenated trajectories of Rec ranged between 0.0006 and 0.1244, those for NCS1 ranged between 0.0150 and 0.1597, while the average c_1_ of the five replicas ranged between 0.4670 and 0.5792 for Rec and between 0.1421 and 0.3302 for NCS1. The decrease of c_1_ values from the average of the single replicas to that of the concatenated trajectories suggests that the longer timescale allows for a more significant and exhaustive conformational sampling.

The RMSIP of the first 20 PC of the ES highlighted a substantial overlapping, and therefore reproducibility, of the conformational subspaces sampled by each individual replica and by the concatenated 1 μs trajectories, as shown by RMSIP values for NCS1 and Rec, ranging from 0.763 to 0.895 (Figure S1), and from 0.665 and 0.828, respectively (Figure S2).

LDA was performed on the projection of MD trajectories along PC1 and PC2 for each protein state. Results showed that for each couple of replicas in all NCS1 (Figure S3) and Rec (Figure S4) states there was at least one conformation that could belong to two different replicas, thus allowing these replicas to be treated as one concatenated trajectory.

Overall, all results confirm that individual 200 ns MD replicas are independent and consistent representations of the dynamic structural behavior of both Rec and NCS1 in their different signaling states. Moreover, concatenated 1 μs trajectories exhaustively sample each protein conformational space and can be subjected to further detailed PSN analyses.

### Time-Persistent Non-covalent Interactions Involved in Intra- and Inter-molecular Communication Define Protein-Specific Structural Networks

Non-covalent interactions are crucial for defining protein three-dimensional structure, yet hydrophobic, electrostatic, and H-bond interactions can have a transient or more persistent essence, depending on whether they are involved in structural information transfer or not. Therefore, we monitored the persistence of these interactions throughout the concatenated 1 μs trajectories of Rec and NCS1 signaling states and determined their significance in shaping the specific PSN (Vendruscolo et al., 2002; Atilgan et al., 2004; Papaleo, 2015) for each protein state. In the resulting graph residues are nodes and persistent interactions represent edges.

Dynamic information from 1 μs MD simulations was encoded in a static PSN (see Methods) and the analysis of graphs identified the presence of key residues (hubs) persistently interacting with many close-contact amino acids (Tables S1–S3). To discriminate whether the identified interactions were significant or transient, p_*T*_ was calculated for each trajectory to filter out short-lived interactions (see Methods) and was found to range between 15.7 and 17.6 for Rec states and between 36.5 and 37.2 for NCS1 variants.

### PSN Analysis Identifies Peculiar Residues With a Key Role in Intramolecular Information Transfer

To assess which ones among the interface residues are responsible for transmitting structural information regarding the Ca^2+^-loading state to their target, the CR index was calculated for each residue of NCS1 (Figure S5) and Rec (Figure S6) target interfaces with each respective Ca^2+^-coordinating Glu residues chosen as representative for each EF-hand motif.

**Figure 1:**
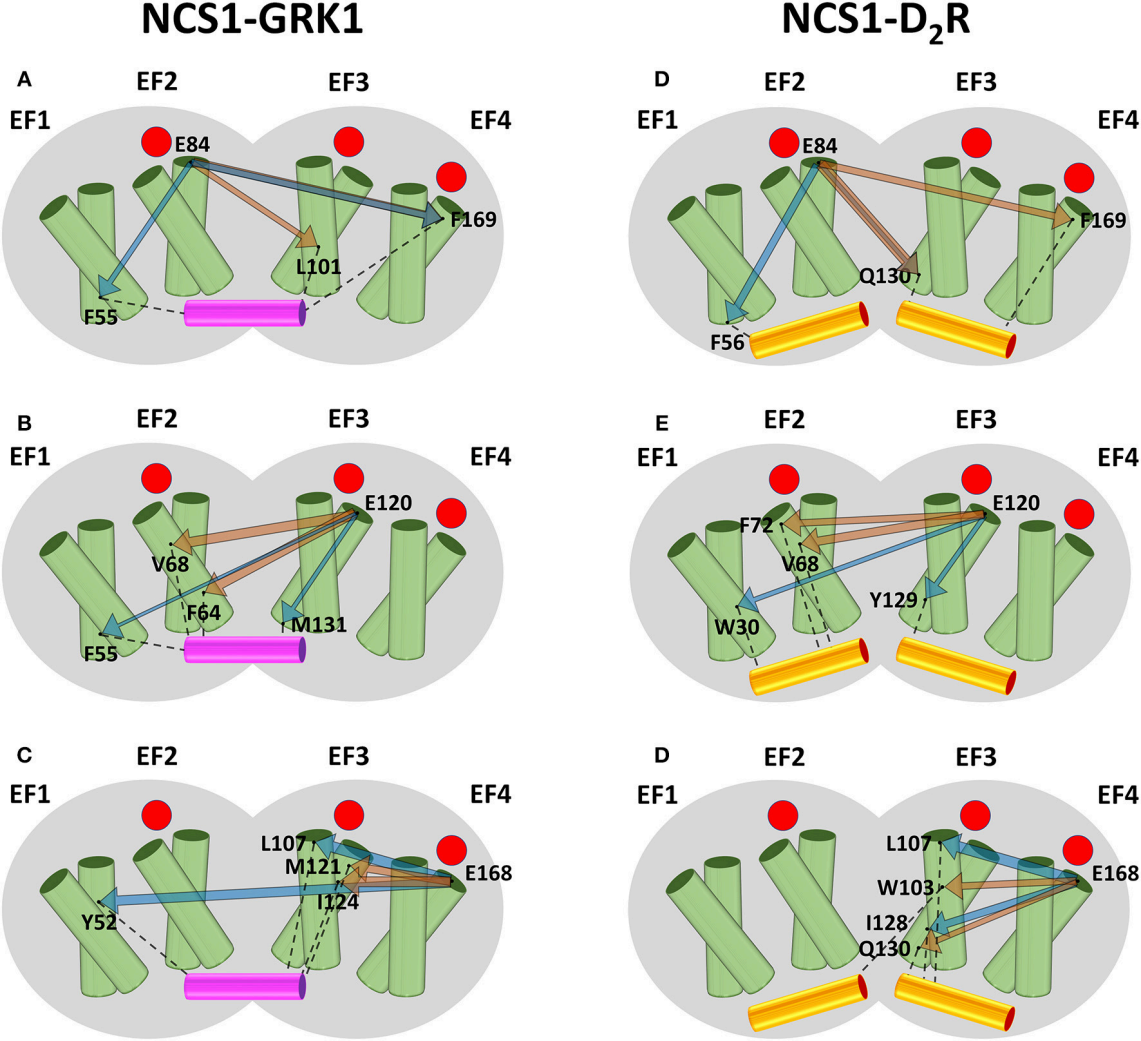
Schematic representation of the intramolecular communication between NCS1 EF-hands and GRK1 (left) or D_2_R (right) interface residues. NCS1 shape is represented in gray, helices of the four EF-hands are represented by green cylinders where the entering helix is in front of the exiting helix, GRK1 and D_2_R peptides are shown in purple and yellow cylinders, respectively. Occupied Ca^2+^-binding sites are represented as red circles. For each peptide, residues representing EF2 **(A,D)**, EF3 **(B,E)** and EF4 **(C,F)**, and the two residues with highest CR (Figure S3) are shown as black dots and labeled. Interactions between interface residues and target peptides are represented by black dashed lines. The communication between EF-hand representatives and interface residues in the absence and in the presence of targets is shown, respectively, by orange and blue arrows, whose width is proportional to CR values (Figure S3).

Results for NCS1, summarized in Figure 1, highlight that in the absence of target peptides (orange arrows) there is a long-range communication from EF2 (Figures 1A,D) to both EF-hands of the C-domain. In detail, L101 (Figure 1A) and Q130 (Figure 1D) belong to the entering (hE) and exiting (hF) helix of EF3, respectively, and F169 (Figures 1A,D) is located at helix hF of EF4 (hF4). On the other hand, communication from EF3 (Figures 1B,E) and EF4 (Figures 1C,F) is overall short-ranged in the absence of the target, reaching for both interfaces only residues located in adjacent EF2 or EF4. Specifically, E120 communicates with F64 and V68 (Figure 1B) or with V68 and F72 (Figure 1E), which are all located on helix hE2. The EF4 representative residue E168 exhibits robust communication with M121 and I124 of helix hF3 (Figure 1C) or W103 and Q130, located, respectively, on helix hE3 and hF3.

In the presence of GRK1 (blue arrows) all three EF-hands show allosteric intradomain properties: EF2 shows robust communication with F55 of helix hE1 and F169 of helix hF4. (Figure 1A); EF3 exhibits high CR values with M131 of helix hF3 and to a lower extent with F55 of helix hE1; finally, EF4 robustly communicates with L107, located on the adjacent helix hE3, and with Y52 of helix hF1.

The pattern of structural information transfer appears different in the presence of another biological target. Overall, communication of NCS1 EF-hands with D_2_R interface travels shorter distances (Figures 1D–F, blue arrows). In detail: EF2 communicates with hE1 residue F56 and hE3 residue Q130 (Figure 1D); EF4 communicates with hE3 residue L107 and hF3 residue I128 (Figure 1F); finally, EF3 communication reaches Y129, also belonging to hF3 helix, and the distant W30 of helix hE1, the only residue exhibiting long-range communication (Figure 1E). In summary, allosteric communication between EF-hands and target interface is a peculiar feature of NCS1-GRK1 complex and of the EF2 motif of NCS1-iso, while robust communication is on average shown on a mid-to-short range by NCS1-iso EF3 and EF4 and by NCS1- D_2_R complex.

**Figure 2:**
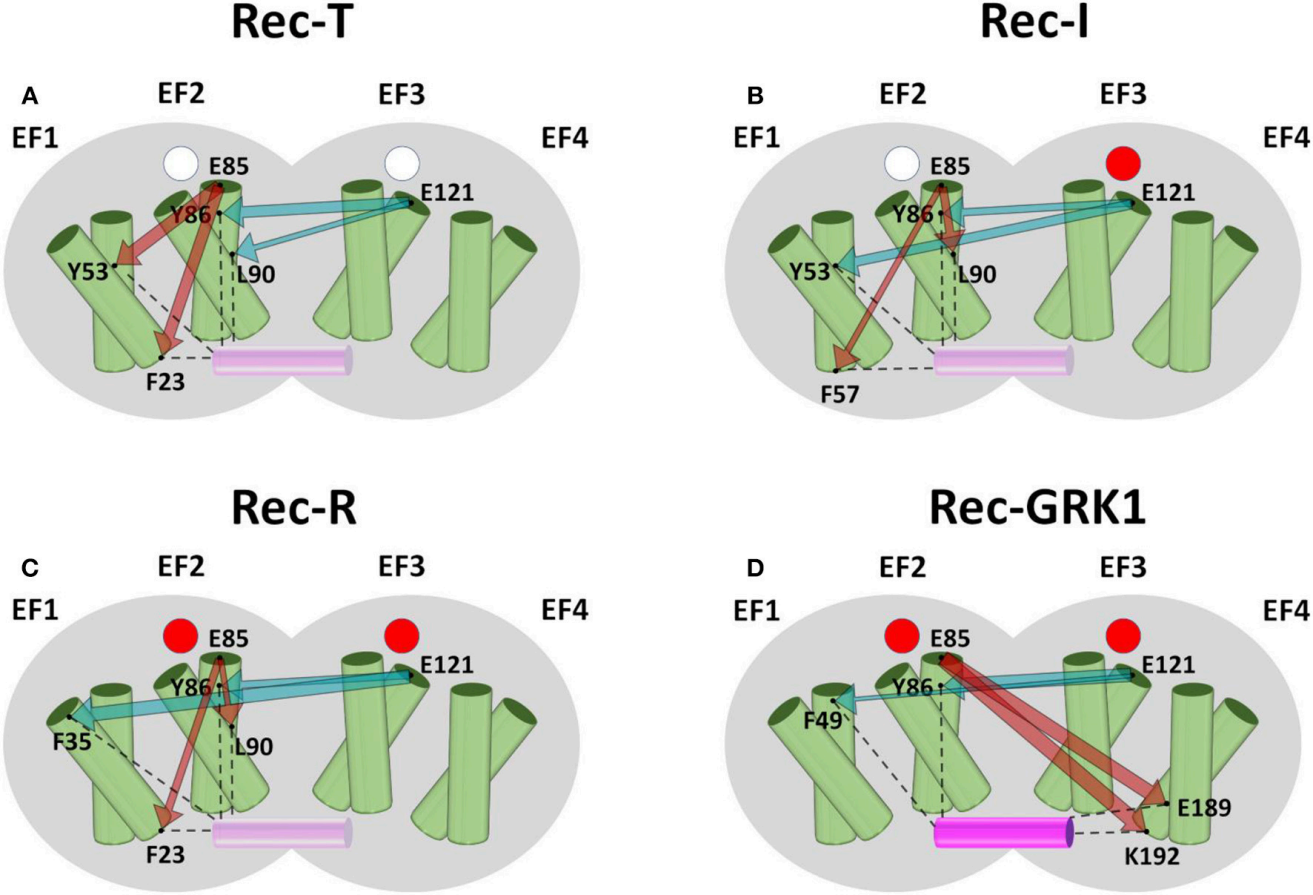
Schematic representation of the intramolecular communication between Rec EF-hands and GRK1 interface residues. Rec shape is represented in gray, helices of the four EF-hands are represented by green cylinders where the entering helix is in front of the exiting helix, GRK1 is shown as a solid or transparent purple cylinder depending on its presence. Empty Ca^2+^ binding sites are represented as white circles, occupied Ca^2+^-binding sites are represented as red circles. Panels show Rec-T (apo, **A**), Rec-I (EF3-Ca^2+^, **B**), Rec-R (Ca^2+^-loaded, **C**) and Rec-GRK1 (Ca^2+^-loaded and peptide bound, **D**). For each case residues representing EF2 and EF3, namely E85 and E121, and the two residues with highest CR (Figure S4) are shown as black dots and labeled. Interactions between interface residues and GRK1 peptide are shown by black dashed lines. The communication between EF-hand representatives and interface residues is represented by dark red (E85) and teal (E121) arrows whose width is proportional to CR values (Figure S4).

Results for Rec, summarized in Figure 2, highlighted robust short-range communication from EF2 to GRK1 interface in the absence of target. In Rec-T (Figure 2A) the absence of Ca^2+^ ions bound to the EF-hands is communicated from EF2 to F23 (hE1) and Y53 (hF1). In Rec-I (Figure 2B), where only EF3 is occupied by a Ca^2+^ ion, the communication of unoccupied EF2 is robust with F57 (hF1) and L90 (hF2). Finally, in Rec-R (Figure 2C), with both EF-hands occupied by Ca^2+^, again EF2 communicates with hE1 residue F23 and hF2 residue L90.

The presence of the GRK1 target changed somewhat the scenario. Rec-GRK1 complex, indeed, revealed a strikingly high allosteric communication between EF2 (E85) and hF4 residues E189 and K192 (Figure 2D), a residue previously identified as crucial for the interaction with GRK residue F3 (Zernii et al., 2011). Long-range communication was observed also when originated in EF3, as shown by the high CR exhibited by Rec-T L90, located in hF2 (Figure 2A), the previously mentioned Y53 in Rec-I (Figure 2B), hE1 residue F35 in Rec-R (Figure 2C), and hF1 residue F49 in Rec-GRK1 complex (Figure 2D). Surprisingly, regardless of the presence of Ca^2+^ or GRK1, EF3 showed a significant robust communication with the first residue of helix hF2, namely Y86. Moreover, Y86 counterpart in NCS1 (F85) was identified as one of the most robustly communicating interface residues with EF2 and EF4, particularly in the absence of targets (Figure S5).

The N-terminal myristoyl group of Rec was also found to robustly communicate with GRK1 interface residues (Figure S7A) in the absence of Ca^2+^ (Rec-T), but this robustness decreased on average upon Ca^2+^-binding to EF3 (Rec-I). Moreover, no CR index could be calculated upon Ca^2+^-binding to EF2 (Rec-R and Rec-GRK1) due to the extrusion of the fatty acid from the hydrophobic crevice via myristoyl switch mechanism (Tanaka et al., 1995), which prevented any persistent interaction with the protein.

Finally, CR index was calculated among EF-hands of Rec and NCS1 (Figure S7B), highlighting substantial medium-range information transfer between EF2 and EF3 of Rec only in the absence of Ca^2+^. NCS1, on the other hand, showed no significant communication between adjacent EF-hands, but the NCS1-GRK1 complex exhibited a surprisingly high robustness in the allosteric communication between EF2 and EF4 (Figure S7B).

### Intermolecular Communication Pathways Between Ca^2+^ Binding Sites and Common Target

Intermolecular communication between GRK1 and EF-hands of Rec and NCS1 was assessed via CR index to compare how the same target could be regulated by two proteins with overlapping interfaces. Results (Figure S8) highlighted that GRK1 residue L6 communicated specifically with NCS1-EF4, T8 with NCS1-EF3, A11 with Rec-EF3 and S13 with NCS1-EF2. Interestingly, residue N12 of GRK1 was found to communicate with both Rec-EF2 and NCS1-EF3, while F15 with EF2 in NCS1 and EF3 in Rec. Overall, the analysis highlighted that the robustness of NCS-target interaction is highly protein-dependent in terms of specificity of the signaling state.

**Figure 3:**
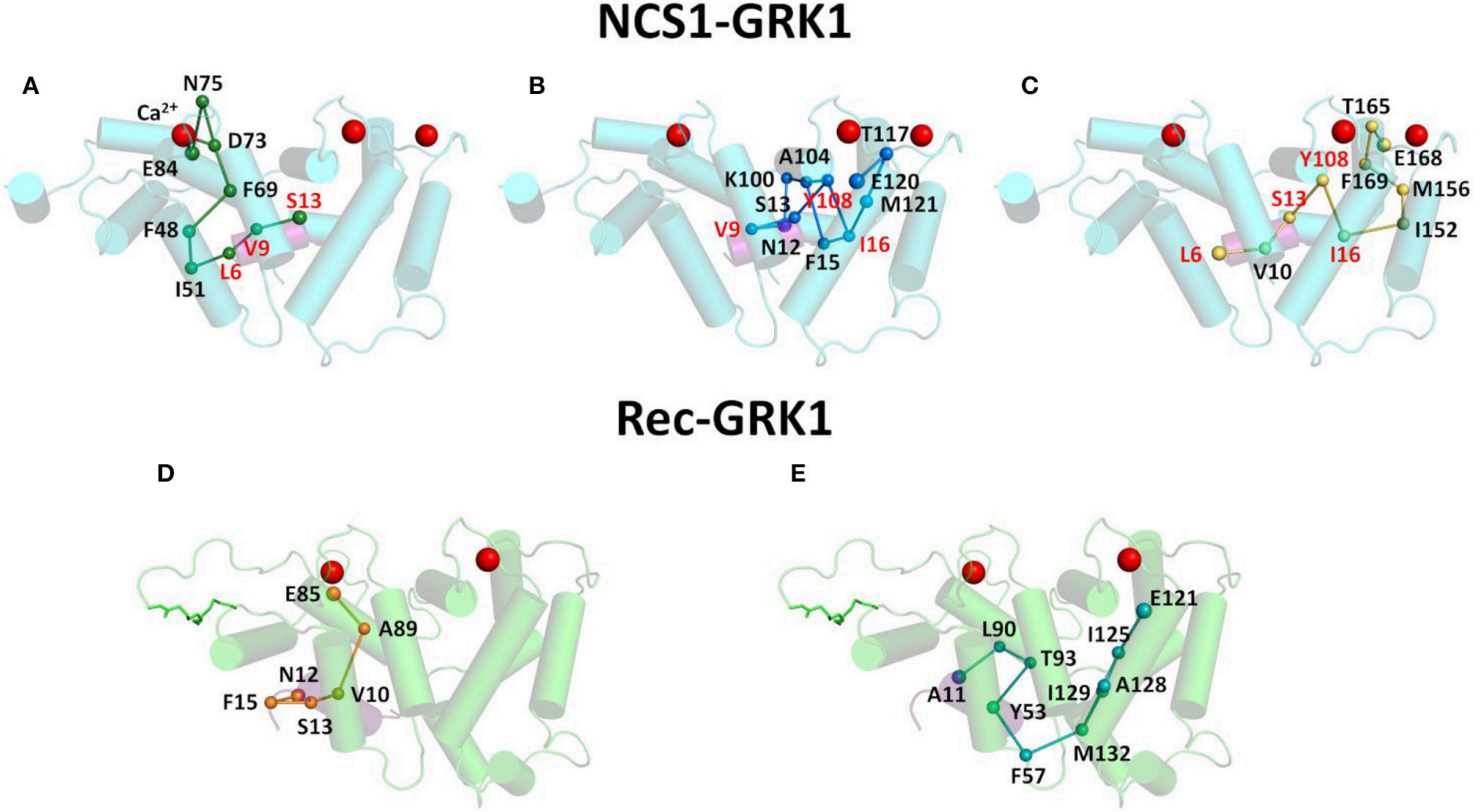
Intermolecular communication pathways between GRK1 peptide and EF-hands of NCS1 (top panels) and Rec (bottom panels). Ca^2+^ ions are shown as red spheres, NCS1, Rec and GRK1 structures are shown in cylindrical cartoon and colored in cyan, light green and purple, respectively, Rec myristoyl group is represented in light green sticks. Pathways with the highest cumulative SB connecting EF-hand representatives and GRK1 residues with the highest CR are shown in sticks and spheres. In detail, pathway connecting NCS1 E84 and GRK1 S13 **(A)** is shown in dark green, pathway connecting NCS1 E120 and GRK1 N12 **(B)** is shown in blue, pathway connecting NCS1 E168 and GRK1 L6 **(C)** is shown in yellow, while pathways connecting Rec E85 and GRK1 N12 **(D)** and Rec E121 and GRK1 A11 **(E)** are shown in orange and teal, respectively. Pathway residues are labeled in black, residues shared by two pathways are labeled in red, GRK1 residues are underlined.

In addition, the previously mentioned GRK1 residues with highest CR were investigated from a topological standpoint concerning the most probable PSN pathways that would allow information transfer to/from EF-hands. The most probable pathways were chosen according to the highest collective SB, nevertheless in some cases multiple pathways with the same score were reported (Figure 3). Pathway analysis (Figure 3) indicated that the pathway from NCS1 EF2 to S13 (Figure 3A; Video V1) included Ca^2+^ ion and that the three GRK1 residues of the pathway, namely L6, V9 and S13, were also involved in the communication of the peptide with NCS1 EF3 and EF4. The pathway from NCS1 EF3 to N12 was the most branched out (Figure 3B; Video V2) with two forks and a double intermolecular communication required to correctly route Ca^2+^-binding information to V9. In addition, EF3-N12 pathway shared the GRK1 residues V9 and S13 with EF2-S13 pathway and both GRK1-I16 and NCS1-Y108 residues with I16 EF4-L6 pathway (Figure 3C; Video V3). Pathway EF4-L6 shared GRK1 residue L6 with EF2-S13 pathway.

The same pathway analysis performed on Rec EF2 with GRK1-N12 (Figure 3D; Video V4) and EF3 with GRK1-A11 (Figure 3E; Video V5) suggested that each EF-hand independently communicates with the target, with no overlapping of the pathways.

Overall, PSN pathway analysis indicated that intermolecular information transfer concerning Ca^2+^-binding was routed by selective pathways connecting EF-hands with GRK1.

### Evolutionary Conservation of Hub Residues Identified in PSN

The connectivity of a PSN is an interesting feature that can be used to compare different states of the same protein and to identify hub residues (Tables S1–S3), that is amino acids involved in persistent interactions with many other residues, therefore playing a key role in maintaining network topology (Raimondi et al., 2011; Fanelli et al., 2013; Marino and Dell'orco, 2016).

**Figure 4:**
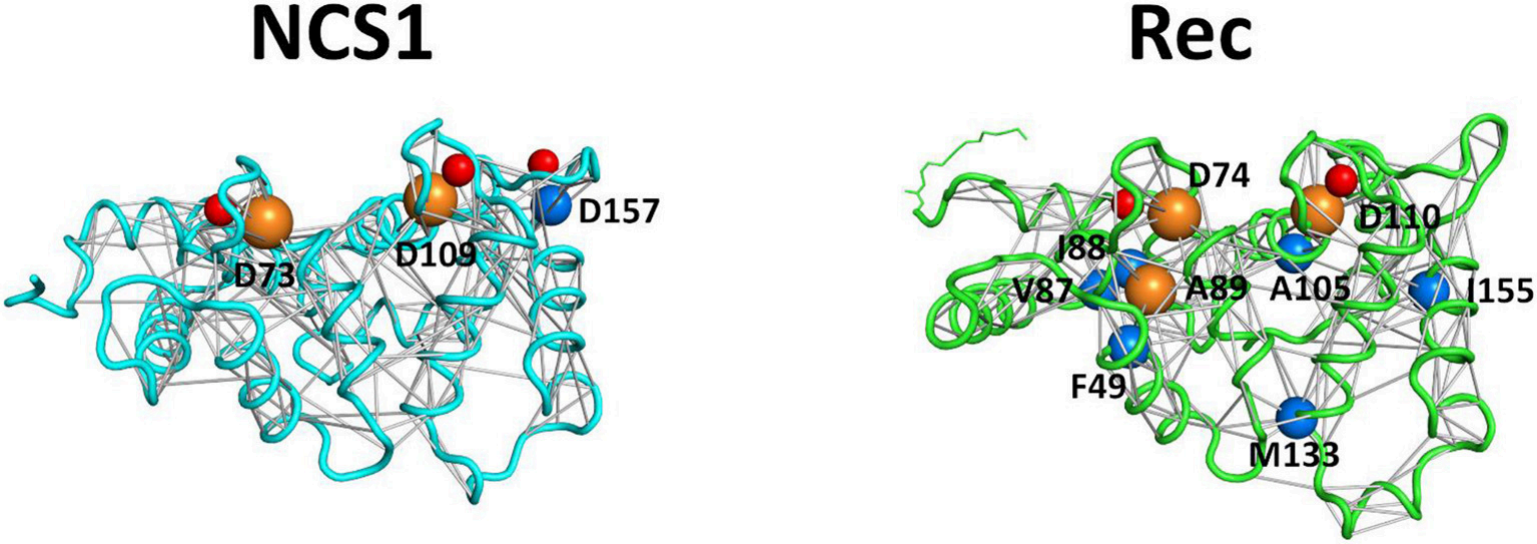
Protein Structure Networks of Ca^2+^-loaded NCS1 (left) and Rec (right) and hub identification. Protein secondary structures are shown as cyan (NCS1) and light green (Rec) cartoons, Ca^2+^ ions are shown as red spheres, Rec myristoyl group is represented in light green sticks. Non-covalent interaction (edges) between residues (nodes) is represented as gray sticks connecting residues Cα or Ca^2+^ ions. Cα atoms are shown as spheres with radius size proportional to the number of connections and colored in blue (degree 7 hubs) or orange (degree 8 hubs).

An example of PSN is represented in Figure 4, where all persistent interactions in Ca^2+^-loaded NCS1 and Rec are shown. Although the number of connections (and consequently hubs) identified in NCS1 and Rec are different due to intrinsic protein properties, the highest degree hubs (degree = 8) in both proteins share their function, as NCS1 D73 and Rec D74 are the first Ca^2+^-coordinating residues of EF2 Ca^2+^-binding loop, while NCS1 D109 and Rec D110 are the equivalent for EF3.

Hub analysis was performed for all NCS1 (Table S1) and Rec (Tables S2, S3) states and was compared to our previously published data (Marino and Dell'orco, 2016) on EF3-Mg^2+^ and Ca^2+^-loaded human GCAP1 (Table S5, and Table S4 in the reference).

**Figure 5:**
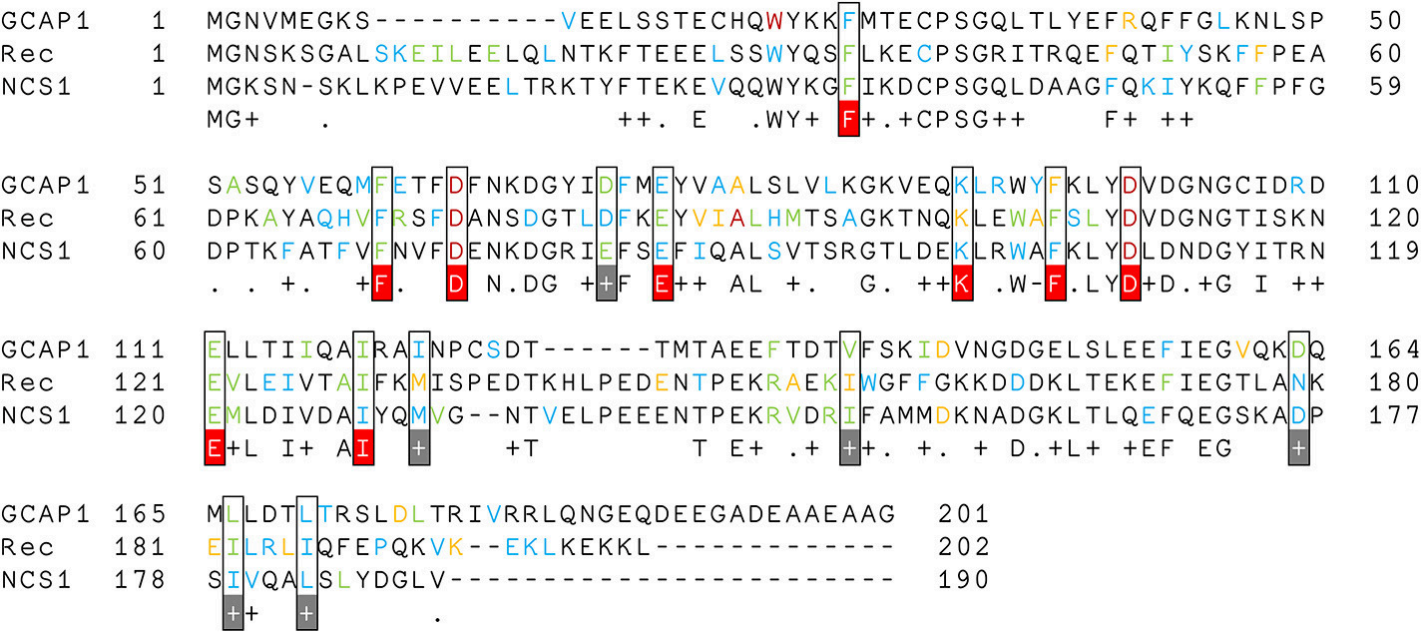
Multiple Sequence Alignment of Rec, NCS1, and GCAP1. Hub residues are colored according to their degree; therefore degree 8 hubs are in dark red, degree 7 hubs are in orange, degree 6 hubs are in green and degree 5 hubs are in blue. Hub residues with degree ≥ 5 in all three proteins are framed and colored in white, conserved residues are highlighted in gray, identical residues are highlighted in red.

Multiple Sequence Alignment (Figure 5) showed that these three NCS proteins have 9 identical and 6 conserved hubs whose maximum degree was at least 5 in any of the simulated states. Interestingly, 4 identical hubs represent the first and the last residue of the Ca^2+^-binding loops of EF2 and EF3, implying that any mutation of these residues could have a detrimental effect on protein structure/function relationship, as previously demonstrated (Kitiratschky et al., 2009; Dell'orco et al., 2010; Vocke et al., 2017; Marino et al., 2018).

**Figure 6:**
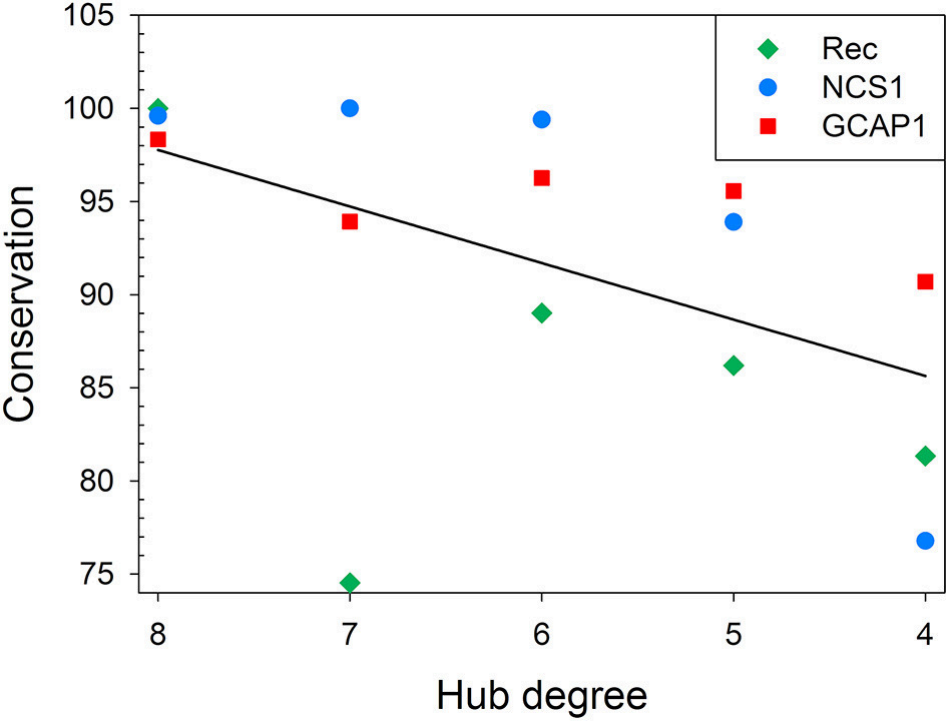
Correlation between hub degree and average residue conservation in UniRef50 clusters for Rec, NCS1, and GCAP1. Average residue conservation in UniRef50 clusters is plotted against hub degree for Rec (green diamonds), NCS1 (blue circles), and GCAP1 (red squares). Residue conservation in UniRef50 clusters shown in Figure 6 and in Tables S1–S3,S5 was calculated as the ratio between the number of sequences where the residue of the seed sequence was conserved and the total number of sequences in the cluster. When a residue was a hub in different signaling states, even with a different degree, only one occurrence of the residue was considered for the calculation of the average of given hub degree. Linear regression of average residue conservation is shown as a black line.

Finally, multiple sequence alignment was run for each protein with their respective UniRef50 cluster (proteins sharing at least 50% s.i. and 80% sequence coverage), and the conservation of each hub residue among homolog proteins was calculated (Tables S1–S3, S5) and compared to their degree (Figure 6). While no apparent mathematical relationship could describe this behavior in general, a clear, protein-specific trend could be identified. All three proteins reported a decrease of the average conservation concurrent with the decrease of the hub degree, with a 62% probability to be linearly correlated. Although the *R*^2^ value of 0.62 is not sufficiently high to clearly infer a correlation between the average conservation and the hub degree, it has to be noticed that there is an intrinsic limitation in the number of points in the x-axis, as the hub degree is a discreet quantity. Therefore, *R*^2^ value is greatly affected by the presence of some poorly conserved (<50%) high degree hub residues of Rec, namely I155, V87, E181, and K194.

## Discussion

NCS1, Rec, and GCAP1 belong to a highly conserved family of proteins called NCS, which are able to specifically regulate a limited number of biological targets in a Ca^2+^-dependent manner. Such homologous proteins share a very similar fold despite a relatively low s.i., specifically: (human) NCS1 and (bovine) Rec have a 44% s.i. (Figure S9A), NCS1 and (human) GCAP1 have a 32% s.i. (Figure S9B) and Rec and GCAP1 have a 31% s.i. (Figure S9C).

The higher s.i. shown by Rec and NCS1 with respect to that exhibited with GCAP1 is probably related to their structural similarities, also reflected in the regulation mechanisms. As previously reported, Rec and NCS1 undergo a Ca^2+^-dependent conformational change known as myristoyl-switch (Ames and Lim, 2012), where the fatty acid chain is extruded from a highly hydrophobic crevice upon Ca^2+^-binding, allowing target interfaces to be solvent-exposed. On the other hand, the myristoyl group in GCAP1 is buried regardless of [Ca^2+^] and mediates communication between N- and C-domains (Marino and Dell'orco, 2016) via the mechanism known as myristoyl-tug (Peshenko et al., 2012). In addition, the three NCS proteins analyzed in the present study have intrinsically diverse structural dynamics, as assessed by the significantly different p_T_ values exhibited by each protein state, which may be ascribable to the specificity of their targets. The only common target is in fact GRK1, shared by NCS1 and Rec.

Analysis of the PSN deriving from thorough MD simulations has been proven to be a valuable tool for the investigation of allosteric properties of NCS proteins (Marino and Dell'orco, 2016). Robust long-range communication from functional EF-hands to target interface was exhibited by both NCS1-GRK1 (Figure 1, left), Rec-GRK1 (Figure 2), and to a lesser extent by NCS-D_2_R (Figure 1, right) complexes. The NCS1-GRK1 complex showed also a strikingly robust communication (Figure S7B) between the C-terminal low affinity Ca^2+^-binding site EF4 (Bourne et al., 2001; Aravind et al., 2008) and the N-terminal high affinity EF2, similarly to what was previously reported for GCAP1 (Marino and Dell'orco, 2016).

**Figure 7:**
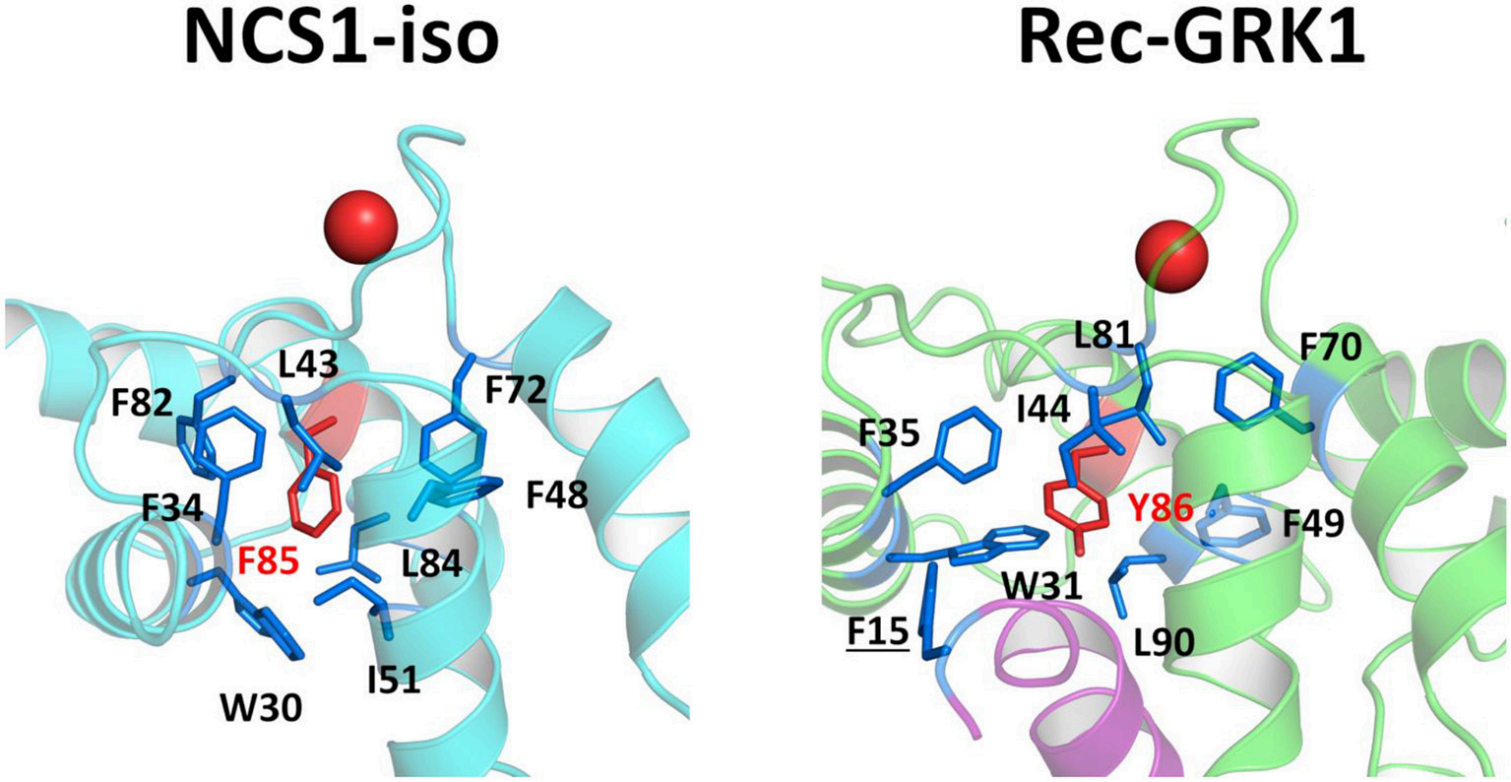
Structural insights into hydrophobic interaction network involving NCS1-iso residue F85 (left) and Rec-GRK1 residue Y86 (right). Protein secondary structures are shown as cyan (NCS1), light green (Rec) and purple (GRK1) cartoons, Ca^2+^ ions are shown as red spheres. Residues F85 and Y86 are represented as red sticks and labeled, hydrophobic residues surrounding F85 or Y86 are represented as blue sticks and labeled, residue F15, belonging to GRK1 peptide is underlined.

Interestingly, Rec-Y86 was found to be particularly important for mediating communication as to the cation loading state of EF3 in all signaling states (Figure 2), while its counterpart NCS1-F85 is involved in robust communication between target interface and EF2 and EF4 in the absence of the target (Figures S5A,D,F). The importance of the two evolutionary conserved residues resides in their peculiar spatial location as both residues belong to a network of tightly packed hydrophobic interactions (Figure 7) involving EF1 and EF2, which is eventually responsible for the stabilization of the entire C-terminal domain.

By comparing state-specific PSN (Figure 4) of NCS proteins, we identified evolutionary conserved hub residues (Figure 5), whose degree appears to have a certain degree of correlation with the conservation among homologs (Figure 6). Such residues evidently play an essential role in converting Ca^2+^-sensing information from EF-hands to a conformational switch required for the regulation of the targets (Tables S1–S3). On the other hand, even in the presence of the same biological target, as is the case for the regulation of GRK1 by Rec and NCS1, intermolecular interaction pathways with the targets are substantially different (Figure 3). Specific intra- and inter-molecular networks of communication are therefore chosen for optimizing the unique function of the NCS protein, despite the high conservation of the hub residues with the family.

Hub residues Rec-E85, Rec-E121, NCS1-E84, NCS1-E120, GCAP1-E75, GCAP1-E111, are conserved in all three NCS proteins, which is not surprising since they are directly involved in Ca^2+^-coordination. Interestingly, the NCS1-E120Q mutation was proven to abolish Ca^2+^ binding in the highest affinity site EF3 of NCS1 and subsequently the myristoyl-switch mechanism (Weiss et al., 2000), to impact stability and unfolding profiles (Muralidhar et al., 2005) and ultimately to prevent D_2_ receptor desensitization (Kabbani et al., 2002). Similar conclusions were drawn for Rec-E85Q mutation (Ames et al., 2002), which prevented Ca^2+^-binding to the high affinity site EF2 and impaired myristoyl switch. Other conserved hubs, though, were responsible for correctly routing intermolecular information in either NCS1 and GCAP1 (namely F69/F60, D73/D64, K100/K91, respectively) or GCAP1 and Rec (namely GCAP1-I119 and Rec I129).

Our topological analysis of the PSN resulting from protein-specific dynamics also highlights amino acids that are crucial for the physiological function of GCAP1 and suggests that any mutation in these key positions may lead to dysfunctional states. In fact, we previously identified GCAP1 hub residues that, when mutated, were associated with retinal dystrophies (Marino and Dell'orco, 2016), namely: D100, which is the target of the D100E/G substitutions (Kitiratschky et al., 2009; Dell'orco et al., 2010; Nong et al., 2014); L84, which is the target of the L84F substitution (Kamenarova et al., 2013; Marino et al., 2015a); Y99, associated with the Y99C mutation (Payne et al., 1998; Sokal et al., 1998); E155, associated with the E155A and E155G mutations (Wilkie et al., 2001); I143, found to be mutated in I143T/N (Nishiguchi et al., 2004). Other conserved residues highlighted in the present study, namely L176 and E111, were recently found to be associated with retinal dystrophies (Vocke et al., 2017; Marino et al., 2018). This group of genetic diseases is characterized by a dysregulation of the second messengers Ca^2+^ and cGMP homeostasis caused by different molecular phenotypes (Koch and Dell'orco, 2013; Dell'orco et al., 2014). Most of the identified mutations indeed affect GCAP1 Ca^2+^ affinity, making such mutants unable to correctly inhibit the target (retinal guanylate cyclase 1 mostly) upon light detection. The decreased Ca^2+^-affinity is either caused by mutations directly involved in Ca^2+^-coordination, as is the case for D100, E111, E155, or by structural effects of mutations, either neighboring ion-coordinating EF-hand loops, such as Y99 and I143, or spatially distant like L84 and L176.

In conclusion, our unbiased PSN analysis highlighted the presence of disease-associated residues in the ensemble of the most robust communication pathways ensuring the correct conformational switch of GCAP1, suggesting a detrimental effect of the point mutation for the protein dynamics under physiological conditions. Thorough topological and dynamic analyses such as the ones performed here could be therefore extended to other cases and eventually they could help understanding at high level of resolution the molecular basis of diseases affecting key protein-protein interactions in a signaling pathway.

## Data Availability

The datasets generated for this study are available on request to the corresponding author.

## Author Contributions

VM and DD conceived the study. VM performed MD simulations and data analysis, and wrote the manuscript with contributions from DD.

### Conflict of Interest Statement

The authors declare that the research was conducted in the absence of any commercial or financial relationships that could be construed as a potential conflict of interest.

## Abbreviations

NCS: Neuronal Calcium Sensors
NCS1: Neuronal Calcium Sensor 1
Rec: Recoverin
GCAP1: Guanylate Cyclase Activating Protein 1
MD: Molecular Dynamics
GRK1: Rhodopsin Kinase
D_2_R: D_2_ Dopamine receptor
PSN: Protein Structure Network
CIB2: Calcium and Integrin Protein 2
“tense Recoverin” or Rec-T: Ca^2+^-free Rec
“intermediate state Recoverin” or Rec-I: Rec with one Ca^2+^ bound to EF3
“relaxed Recoverin” or Rec-R: Ca^2+^-loaded Rec
Rec-GRK1: Ca^2+^-loaded Rec bound to GRK1 peptide
“isolated NCS1”or NCS1-iso: Ca^2+^-loaded uncomplexed NCS1
NCS1-D_2_R: Ca^2+^-loaded NCS1 bound to D_2_ Dopamine receptor peptides
NCS1-GRK1: Ca^2+^-loaded NCS1 bound to Rhodopsin Kinase peptide
PCn: n-th Principal Component
ES: Essential Subspace
RMSIP: Root Mean-Square Inner Product
LDn: n-th Linear Discriminant
CR: Communication Robustness
SB: Selective Betweenness
s.i.: sequence identity
hEn: entering helix of n-th EF-hand
hFn: exiting helix of n-th EF-hand
MSA: Multiple Sequence Alignment.
