# Supplementary material for: Evolutionary-Conserved Allosteric Properties of Three Neuronal Calcium Sensor Proteins
Source: Front Mol Neurosci. 2019 Mar 7;12:50. doi: 10.3389/fnmol.2019.00050 (PMC6417375; doi:10.3389/fnmol.2019.00050)
Supplement: Supplementary file 6 [file Data_Sheet_1.pdf]

Supplementary Material

Evolutionary-conserved allosteric properties of three Neuronal Calcium Sensors

Valerio Marino*, Daniele Dell'Orco

*** Correspondence:** Corresponding Author: valerio.marino@univr.it

Supplementary methods

Structural modeling of Rec and NCS1

Ca^{2+}-free Rec ("tense Recoverin", Rec-T) was frame 16 of PDB file 1IKU (Tanaka et al., 1995), EF3-Ca^{2+} Rec ("intermediate state Recoverin", Rec-I) was frame 14 of PDB file 1LA3 (Ames et al., 2002) and Ca^{2+}-loaded ("relaxed Recoverin", Rec-R) was frame 13 of PDB file 1JSA (Ames et al., 1997). Since Rec-I structure was solved with the mutation E85Q that prevented Ca^{2+}-binding to EF2 (Ames et al., 2002), residue Gln 85 was mutated back to WT Glu by in silico mutagenesis. The rotamer was selected according to the best solution proposed by "mutate residue" function of Maestro (Schrodinger).

The structure of Ca^{2+}-loaded Rec bound to GRK1 peptide (Rec-GRK1) was modeled starting from the average structure of PDB file 2I94 (Ames et al., 2006), N-terminus was modeled by superimposition of Cα of residues 11-13 of 2I94 and 1JSA frame 14 (RMSD= 0.014 Å), finally 1JSA 1-10 structure was merged with 2I94 11-189. C-terminus was modeled as reported in (Zernii et al., 2011), briefly: Cα of residues 181-187 of 1OMR (Weiergraber et al., 2003) were superimposed to 2I94 (RMSD= 0.627 Å) and 2I94 1-186 residues were merged with 1OMR 187-202 residues.

Three-dimensional structure for Ca^{2+}-loaded uncomplexed NCS1 ("isolated NCS1", NCS1-iso) was modeled selecting chain B (5AEQ_B) from the PDB file 5AEQ (resolution 1.95 Å) (Pandalaneni et al., 2015) as a template, due to its better completeness with respect to chain A (5AEQ_A). N-terminal missing residues 2-9 were modeled by superimposition of Cα of residues 10-12 of 5AEQ_A and 5AEQ_B (RMSD= 0.025 Å), 5AEQ_A 2-11 residues (up to E11-Cα) were merged with 5AEQ_B 11-185 (from E11-C=O). The missing loop 134-138 was modeled by superimposition of Cα of residues 130-143 of PDB file 1G8I (Bourne et al., 2001) chain B (1G8I_B) and 5AEQ_B (RMSD= 0.063 Å), then IG8I_B 133-140 residues were merged with 5AEQ_B 1-132/141-184. C-terminal missing residues 185-190 of 5AEQ_B were modeled by superimposition of Cα of residues 183-185 of PDB file 1G8I (Bourne et al., 2001) chain A (1G8I_A) and 5AEQ_B (RMSD= 0.054 Å), finally 1G8I_A 184-190 residues (from S184-C=O) were merged with 5AEQ_B 1-185 (up to S184 Cα) to achieve 100% structural coverage.

Ca^{2+}-loaded NCS1 bound to D_2 Dopamine receptor peptides (NCS1- D_2R) was modeled over PDB file 5AER (resolution 2.19 Å) (Pandalaneni et al., 2015) template, where missing residues 2-9 were modeled by superimposition of Cα of residues 10-12 of 5AEQ_A and 5AER (RMSD= 0.037 Å), then 5AEQ_A 2-11 residues (up to E11-Cα) were merged with 5AER 11-190 (from E11-C=O).

Ca^{2+}-loaded NCS1 bound to Rhodopsin Kinase peptide (NCS1-GRK1) was modeled over PDB file 5AFP (resolution 2.3 Å) (Pandalaneni et al., 2015) chain A (5AFP_A) template due to its better completeness with respect to chain B (5AFP_B). N-terminal missing residues 2-7 were modeled by

superimposition of Cα of residues 8-10 of 5AFP_A and 5AEQ_A (RMSD= 0.094 Å), then 5AEQ_A 2-7 residues were merged with 5AFP_A 8-185. Missing loop 134-137 was modeled by superimposition of Cα of residues 131-139 of PDB file 1G8I (Bourne et al., 2001) chain B (1G8I_B) and 5AFP_A (RMSD= 0.467 Å), IG8I_B 130-140 residues were merged with 5AEQ_B 1-129/141-184. C-terminal missing residues 185-190 of 5AFP_A were modeled by superimposition of Cα of residues 182-184 of PDB file 1G8I chain A (1G8I_A) and 5AFP_A (RMSD= 0.021 Å), finally 1G8I_A 184-190 residues were merged with 5AFP_A 1-183 (up to S184 Cα) to achieve complete structural coverage.

Principal Component Analysis and Linear Discriminant Analysis

The cosine content (c_1) (Hess, 2002) of *PC1* was calculated as follows:

$$c_1 = \frac{2}{T}\left(\int_0^T \cos\left(\frac{\pi t}{T}\right) PC1(t)dt\right)^2 \left(\int_0^T PC1^2(t)dt\right)^{-1}$$

Where *T* is the duration of the simulations.

Root-Mean Square Inner Product (RMSIP) was calculated as follows:

$$RMSIP = \left(\frac{1}{S}\sum_{i=1;\, j=1}^{S} \left(v_i^A \cdot v_j^B\right)^2\right)^{\frac{1}{2}}$$

Where v_i^A and v_j^B represent eigenvectors of the ES belonging to replicas *A* and *B*, while *S* is the number of PC in the ES (20 in our case).

Protein Structure Network generation and analysis

Communication Robustness index combines the persistence threshold p_T used to filter out non-bonded interactions from the PSN, the length *l* of shortest paths connecting residues *A* and *B* and their number (σ_{AB}) as follows:

$$CR_{AB} = \frac{\sigma_{AB} \cdot p_T}{l}$$

Selective Betweenness of each residue *v* (*SB*(*v*)) belonging to a pathway is defined as follows:

$$SB(v) = \frac{\sigma_{AB}}{\sigma_{AB}(v)}$$

Where σ_{AB} is the number of shortest paths between *A* and *B* and $\sigma_{AB}(v)$ is the number of shortest paths between *A* and *B* that cross *v*.

Supplementary figures

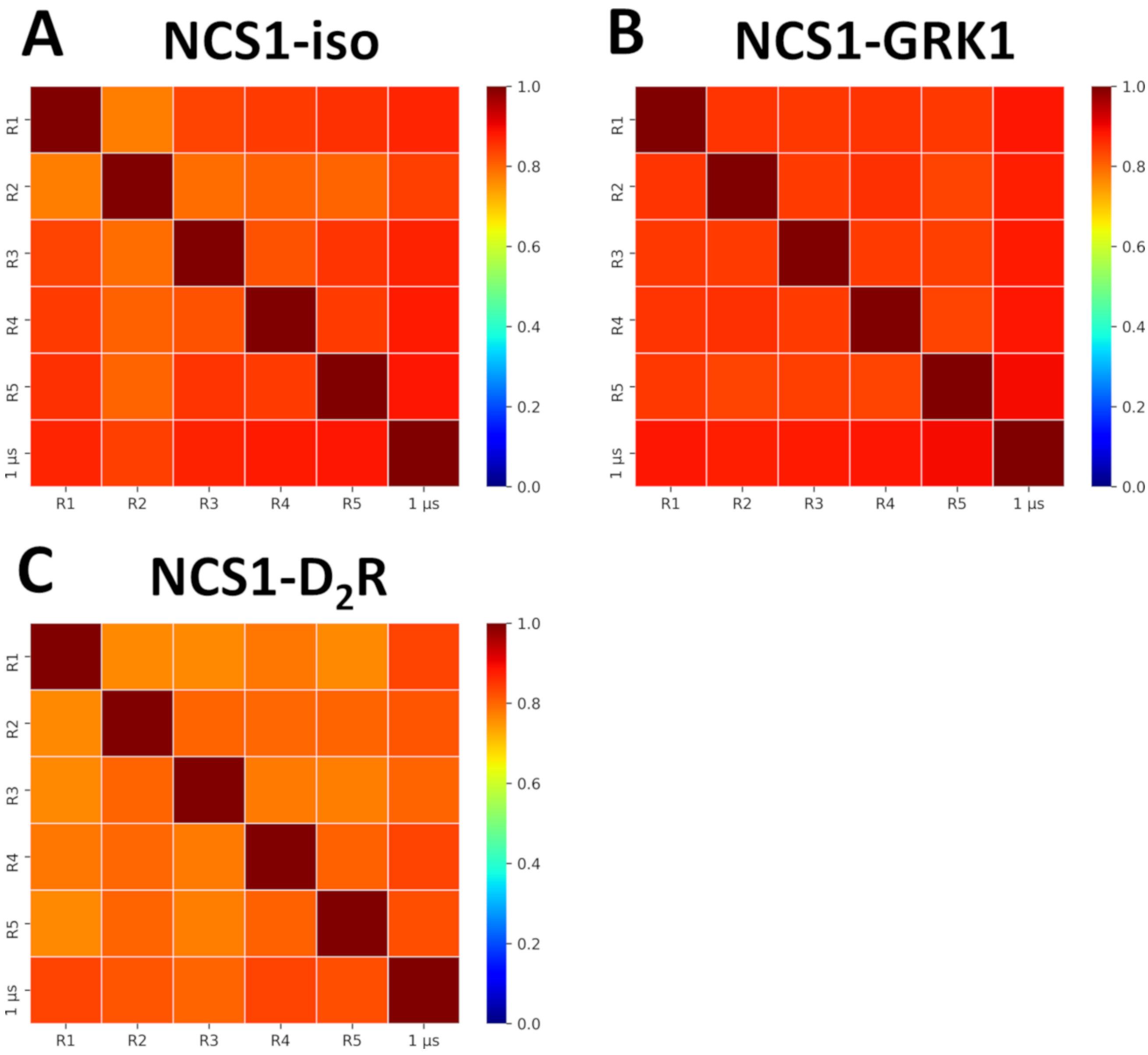

Figure S1. Root-Mean Square Inner Product (RMSIP) of the first 20 PC extracted for each protein from the five 200 ns replicas (R1 to R5) and the concatenated 1 µs trajectories of NCS1. RMSIP was calculated for each replica vs one another and vs concatenated 1 µs trajectory (1 µs) for NCS1-iso (A), NCS1-GRK1 (B), NCS1-D_2R (C). RMSIP values, ranging from 0 to 1, are represented in a color scale from blue to red.

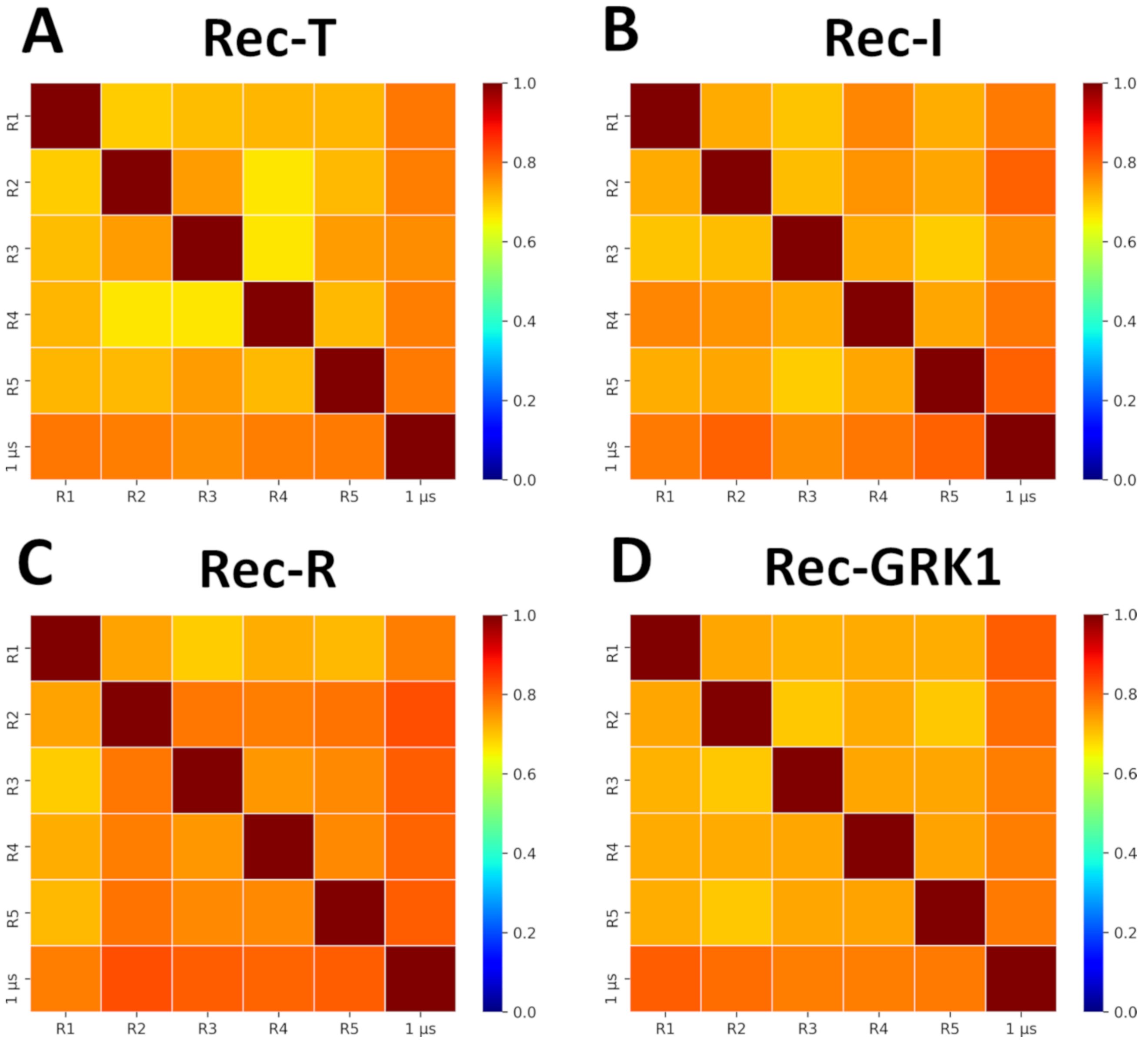

Figure S2. Root-Mean Square Inner Product (RMSIP) of the first 20 PC extracted for each protein from the five 200 ns replicas (R1 to R5) and the concatenated 1 μs trajectories of Rec. RMSIP was calculated for each replica vs one another and vs concatenated 1 μs trajectory (1 μs) for Rec-T (A), Rec-I (B), Rec-R (C), Rec-GRK1 (D). RMSIP values, ranging from 0 to 1, are represented in a color scale from blue to red.

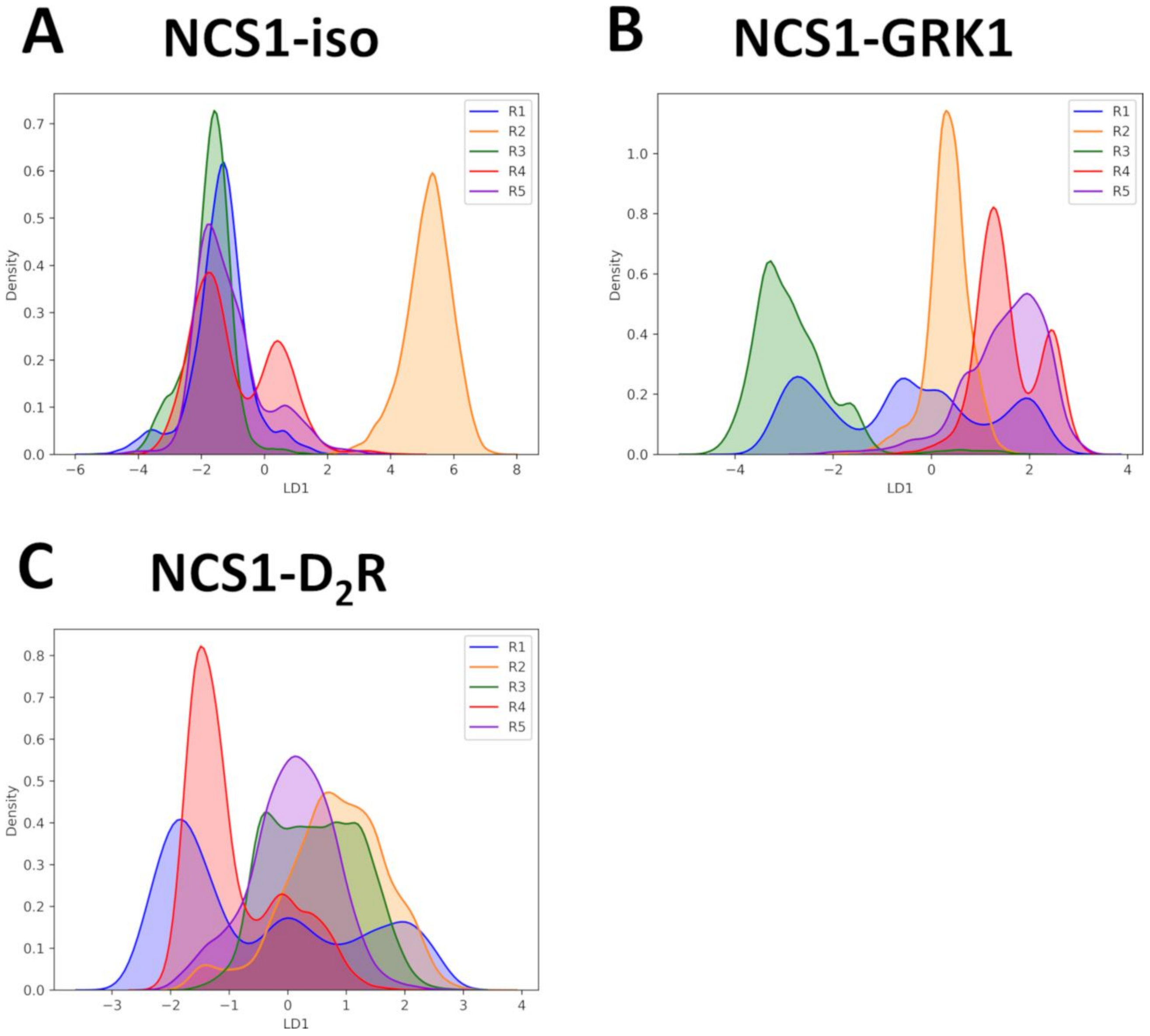

Figure S3. Linear Discriminant Analysis of the projections of the MD replicas on the first two Principal Components extracted from the concatenated 1 μs trajectories of NCS1. Density of frames from R1 (blue), R2 (orange), R3 (green), R4 (red), R5 (purple) replicas of NCS1-iso (A), NCS1-GRK1 (B), NCS1-D_2R (C) projected on LD1. Data were smoothed using Kernel Density Estimation smoothing (see Methods).

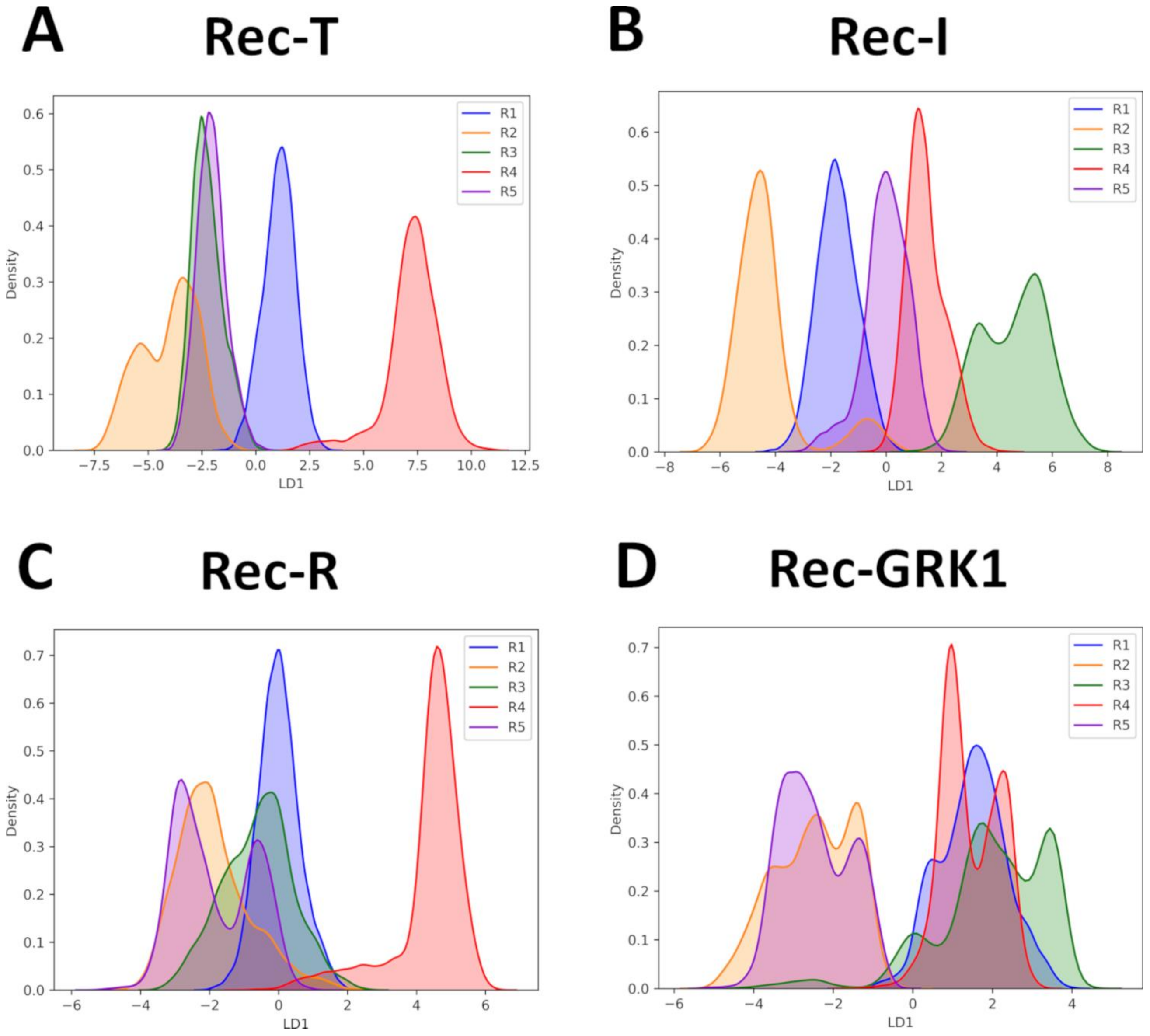

Figure S4. Linear Discriminant Analysis of the projections of the MD replicas on the first two Principal Components extracted from the concatenated 1 μs trajectories of Rec. Density of frames from R1 (blue), R2 (orange), R3 (green), R4 (red), R5 (purple) replicas of Rec-T (A), Rec-I (B), Rec-R (C), Rec-GRK1 (D) projected on LD1. Data were smoothed using Kernel Density Estimation smoothing (see Methods).

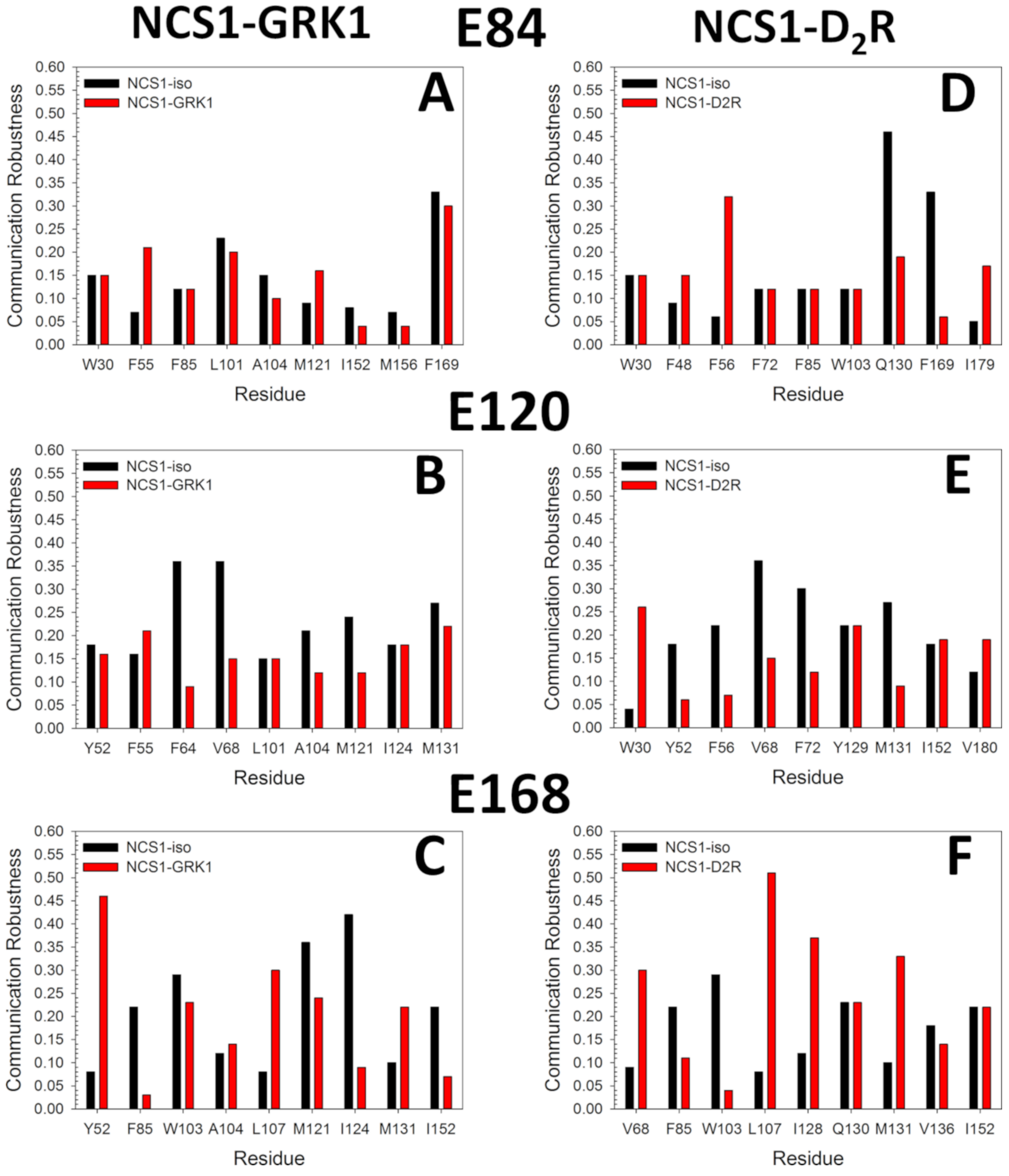

Figure S5. Intramolecular communication between NCS1 EF-hands and GRK1 (left panels) or D_2R (right panels) interface residues. CR index is reported for a subset of 9 NCS1 interface residues representing the 5 highest CR values in at least one state between NCS-iso (black) and NCS1-GRK1 or NCS1-D_2R states (red). CR index was calculated with respect to EF2 representative E84 (panels A and D), EF3 representative E120 (panels B and E) and EF4 representative E168 (panels C and F).

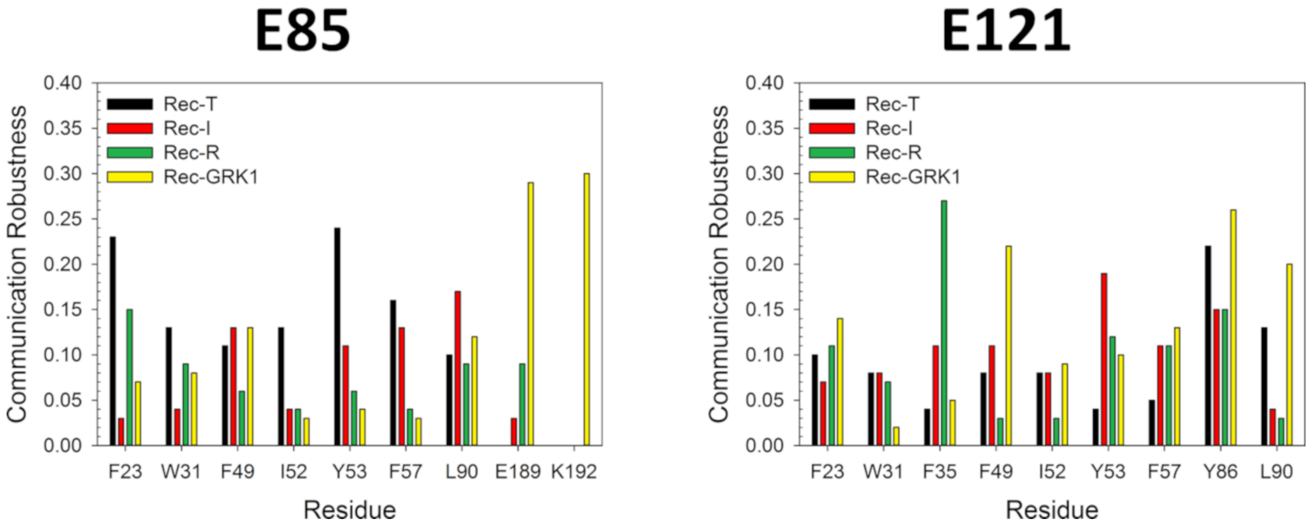

Figure S6. Intramolecular communication between Rec EF-hands and GRK1 interface residues. CR index is reported for a subset of 9 Rec interface residues representing the 5 highest CR values in at least one state between Rec-T (black), Rec-I (red), Rec-R (green) and Rec-GRK1 (yellow). CR index was calculated with respect to EF2 representative E85 (left) and EF3 representative E121 (right).

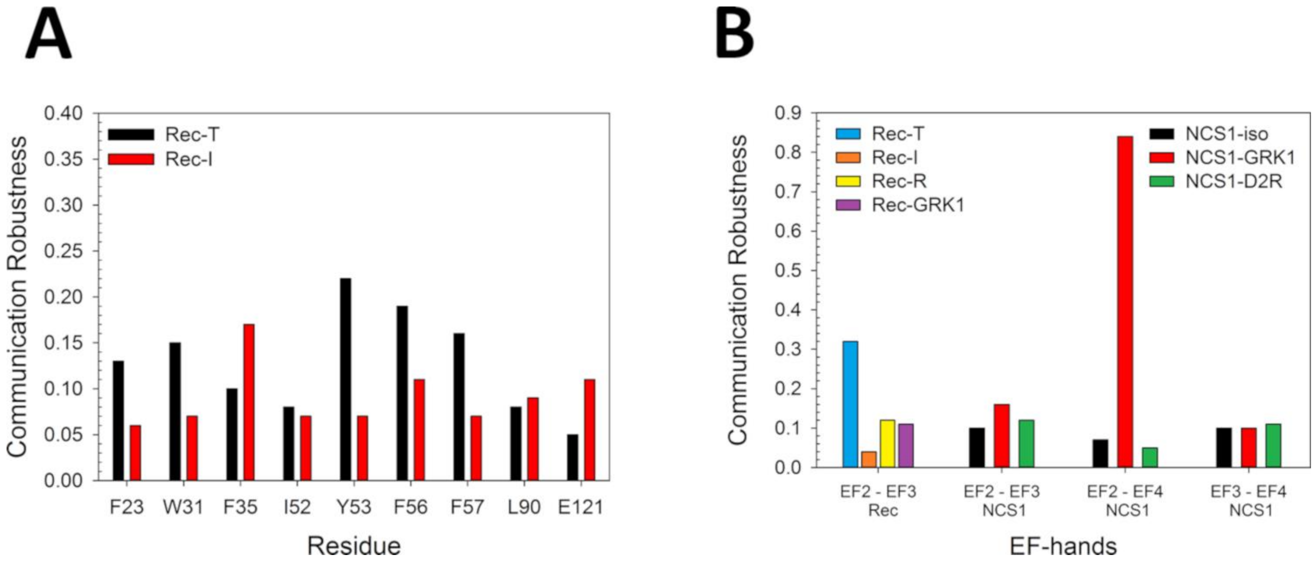

Figure S7. Intramolecular communication between Rec myristoyl group and GRK1 interface residues (A) and among EF-hands of Rec and NCS1 (B). A) CR index, calculated with respect to myristoyl group, is reported for a subset of 9 Rec interface residues representing the 5 highest CR values in at least one state between Rec-T (black) and Rec-I (red); no values are reported for Rec-R and Rec-GRK1 due to the lack of persistent interactions with any interface residue. B) CR index is calculated for Rec-T (blue), Rec-I (orange), Rec-R (yellow), Rec-GRK1 (purple) between E85 and E121 (EF2-EF3), and for NCS1-iso (black), NCS1-GRK1 (red), NCS1-D_2R (green) between E84 and E120 (EF2-EF3), E84 and E168 (EF2-EF4), E120 and E168 (EF3-EF4).

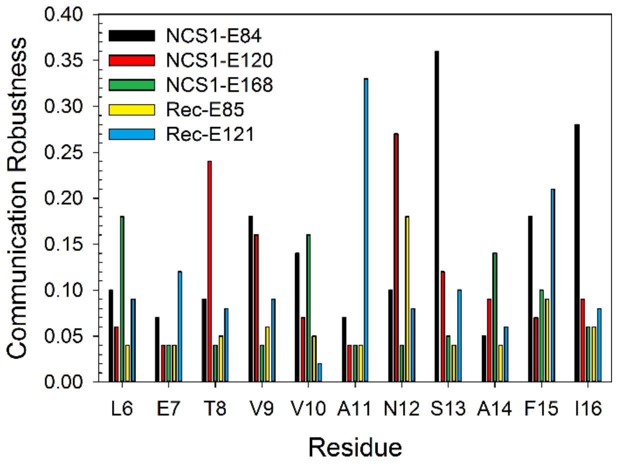

Figure S8. Intermolecular communication between GRK1 and EF-hands of Rec and NCS1. CR index is calculated between NCS1-E84 (black), NCS1-E120 (red), NCS1-E168 (green), Rec-E85 (yellow) and Rec-E121 (blue) and GRK1 residues resolved in both PDB structures (L6-I16).

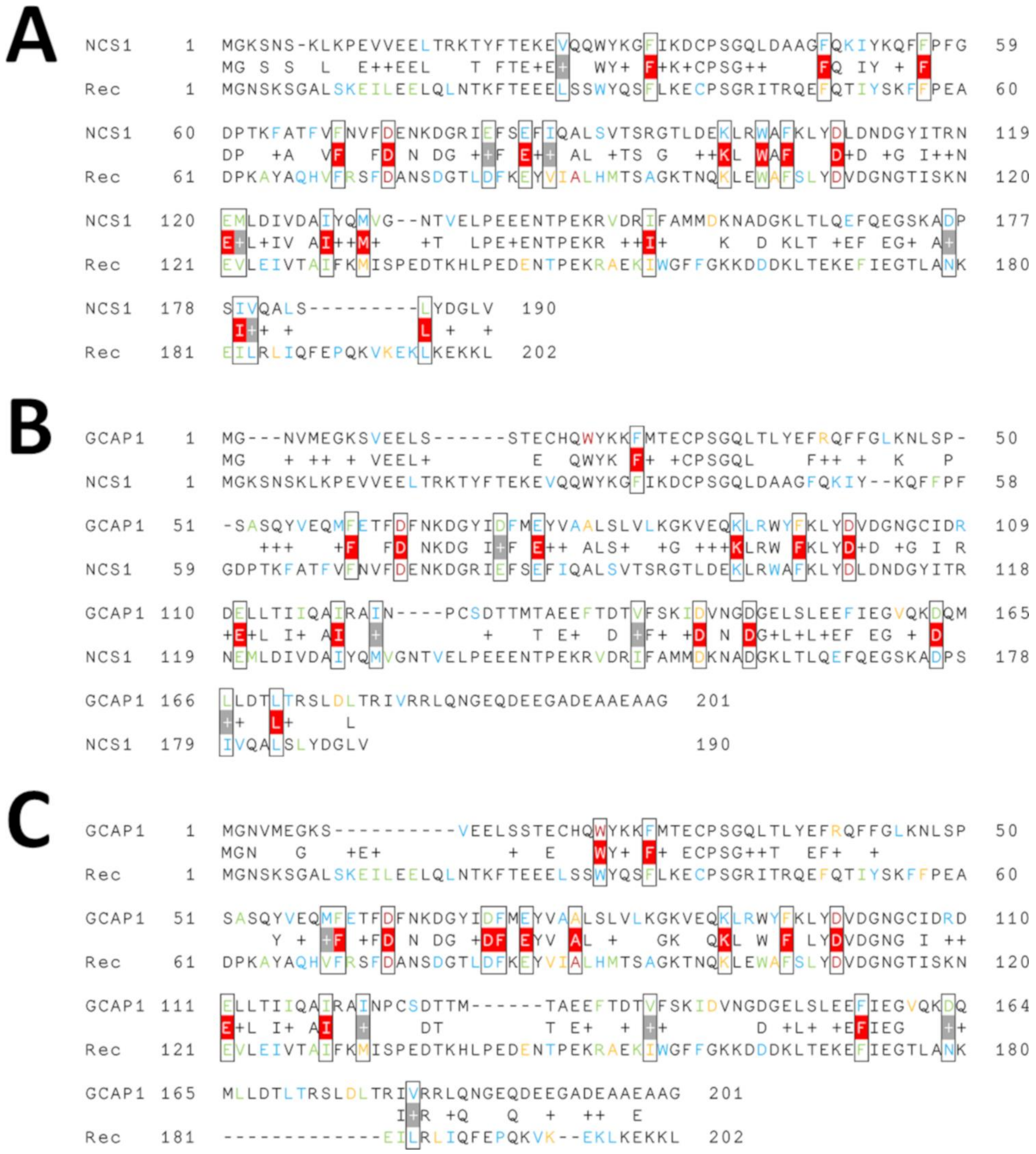

Figure S9. Pairwise global sequence alignment between Rec, NCS1 and GCAP1. Pairwise alignment of A) NCS1 and Rec, B) NCS1 and GCAP1, C) GCAP1 and Rec. Hub residues are colored according to their degree; therefore degree 8 hubs are in dark red, degree 7 hubs are in orange, degree 6 hubs are in green and degree 5 hubs are in blue. Hub residues with degree ≥ 5 in both proteins are framed and colored in white, conserved residues are highlighted in gray, identical residues are highlighted in red.

Video V1. Intermolecular communication pathways between GRK1 peptide and EF2 of NCS1. Ca^{2+} ions are shown as red spheres, NCS1 and GRK1 structures are shown in cylindrical cartoon and colored in cyan and purple respectively. Pathway residues with the highest cumulative SB connecting EF2 representative E84 and the GRK1 residue with the highest CR (S13) are shown in dark green sticks and spheres and labelled.

Video V2. Intermolecular communication pathways between GRK1 peptide and EF3 of NCS1. Ca^{2+} ions are shown as red spheres, NCS1 and GRK1 structures are shown in cylindrical cartoon and colored in cyan and purple respectively. Pathway residues with the highest cumulative SB connecting EF2 representative E120 and the GRK1 residue with the highest CR (N12) are shown in blue sticks and spheres and labelled.

Video V3. Intermolecular communication pathways between GRK1 peptide and EF4 of NCS1. Ca^{2+} ions are shown as red spheres, NCS1 and GRK1 structures are shown in cylindrical cartoon and colored in cyan and purple respectively. Pathway residues with the highest cumulative SB connecting EF2 representative E168 and the GRK1 residue with the highest CR (L63) are shown in yellow sticks and spheres and labelled.

Video V4. Intermolecular communication pathways between GRK1 peptide and EF2 of Rec. Ca^{2+} ions are shown as red spheres, Rec and GRK1 structures are shown in cylindrical cartoon and colored in light green and purple respectively, Rec myristoyl group is represented in light green sticks. Pathway residues with the highest cumulative SB connecting EF2 representative E85 and the GRK1 residue with the highest CR (N12) are shown in orange sticks and spheres and labelled.

Video V5. Intermolecular communication pathways between GRK1 peptide and EF3 of Rec. Ca^{2+} ions are shown as red spheres, NCS1 and GRK1 structures are shown in cylindrical cartoon and colored in light green and purple respectively, Rec myristoyl group is represented in light green sticks. Pathway residues with the highest cumulative SB connecting EF3 representative E121 and the GRK1 residue with the highest CR (A11) are shown in teal sticks and spheres and labelled.

Deg	NCS1	NCS1 - D_2R	NCS1 - GRK1
8	D73 (99.2), D109 (100)	D73 (99.2), D109 (100)	D73 (99.2), D109 (100)
7	D157 (100)	D157 (100)	D157 (100)
6	F34 (98), F69 (99.2), E120 (100), M121 (100)	F56 (99.2), M121 (100), V132 (98.8), L185 (98.8)	E84 (100), M121 (100), V149 (100), I152 (100)
5	L16 (98.4), V27 (10.4), F48 (99.2), K50 (96.8), F64 (99.2), F67 (12.4), E84 (100), S90 (100), F105 (100), I128 (100), M131 (100), V149 (100), E168 (100), D176 (99.6), V180 (100)	L16 (98.4), V27 (10.4), F34 (98), F69 (99.2), E84 (100), I86 (100), S90 (100), F105 (100), E120 (100), I128 (100), M131 (100), V136 (99.2), R148 (100), V149 (100), R151 (100), I152 (100), E168 (100), I179 (100), L183 (100)	L16 (98.4), V27 (10.4), F34 (98), I51 (99.2), F56 (99.2), F67 (12.4), F69 (99.2), S90 (100), K100 (100), W103 (100), F105 (100), E120 (100), I128 (100), M131 (100), R148 (100), E168 (100)
4	V13 (10.8), D37 (98), K53 (13.2), F56 (99.2), K63 (12.4), T66 (11.2), V68 (99.2), N70 (60.8), V71 (99.2), N75 (12.4), D77 (12.4), E81 (11.6), I86 (100), R94 (100), W103 (100), A104 (100), D111 (100), D113 (100), D123 (11.2), I124 (100), A127 (99.6), Y129 (100), R148 (100), I152 (100), M155 (92), M156 (100), D161 (12.4), F169 (100), Q170 (11.2), E171 (98.8), I179 (100)	V13 (10.8), W30 (98.4), G33 (97.2), D37 (98), F48 (99.2), I51 (99.2), Y52 (99.2), F55 (99.2), K63 (12.4), F64 (99.2), F67 (12.4), V68 (99.2), V71 (99.2), N75 (12.4), D77 (12.4), E81 (11.6), F85 (100), L89 (100), S93 (100), R94 (100), A104 (100), D111 (100), D113 (100), R118 (11.2), A127 (99.6), Y129 (100), E142 (99.6), T144 (99.6), M155 (92), M156 (100), D161 (12.4), E171 (98.8), D176 (99.6), V180 (100), L189 (98.8)	V13 (10.8), W30 (98.4), F48 (99.2), F55 (99.2), K63 (12.4), F64 (99.2), V68 (99.2), N70 (60.8), V71 (99.2), D77 (12.4), I86 (100), Q87 (11.2), A88 (100), S93 (100), R94 (100), D111 (100), D113 (100), R118 (11.2), V125 (100), A127 (99.6), V132 (98.8), T144 (99.6), D161 (12.4), F169 (100), E171 (98.8), D176 (99.6), L183 (100)

Table ST1. Hubs for PSN of each NCS1 state. For each degree, hub residues of NCS1 and their conservation in UniRef50 cluster are reported.

Deg	Rec-T					Rec-I				
8						D110 (100)				
7	A105 (100)	I155 (45.1)								
6	L90 (100)	D110 (100)	A128 (82)			F56 (99.2)	M121 (100)	V132 (98.8)	L185 (98.8)	
5	F35 (100)	F56 (99.2)	I88 (84.4)	A89 (100)	W104 (100)	K11 (91)	L19 (84.4)	F35 (100)	F56 (99.2)	F57 (99.2)
	M132 (65.6)	A152 (81.1)	K154 (98.4)	F159 (95.9)	F172 (92.6)	Q67 (64.8)	H68 (100)	L81 (98.4)	F83 (100)	L90 (100)
	N179 (87.7)	I182 (57.4)				A95 (26.2)	W104 (100)	F106 (100)	V122 (92.6)	E124 (97.5)
						A128 (82)	I129 (99.2)	M132 (65.6)	T147 (95.9)	A152 (81.1)
						K154 (98.4)	W156 (96.7)	F159 (95.9)	N179 (87.7)	L185 (92.6)
4	N3 (97.5)	L17 (99.2)	L19 (84.4)	W31 (99.2)	F49 (99.2)	S10 (94.3)	L14 (99.2)	E16 (39.3)	L17 (99.2)	W31 (99.2)
	I52 (99.2)	A66 (98.4)	V69 (100)	F83 (100)	S94 (77.9)	Q50 (35.2)	T51 (8.2)	I52 (99.2)	Y65 (98.4)	A66 (98.4)
	E103 (97.5)	F106 (100)	S107 (89.3)	V122 (92.6)	E124 (97.5)	F70 (100)	F73 (100)	N76 (99.2)	D82 (100)	Y86 (96.7)
	I125 (95.9)	I129 (99.2)	K131 (89.3)	T147 (95.9)	W156 (96.7)	V87 (37.7)	A89 (100)	H91 (96.7)	T93 (99.2)	A105 (100)
	F158 (54.1)					L108 (98.4)	Y109 (51.6)	D112 (100)	K119 (99.2)	I125 (95.9)
						F130 (98.4)	T138 (1.6)	E145 (99.2)	K150 (97.5)	E153 (43.4)
						K161 (96.7)	F172 (92.6)	I173 (88.5)	L183 (88.5)	R184 (87.7)
						I186 (91.8)	Q187 (84.4)			

Table ST2. Hubs for PSN of Rec-T and Rec-I states. For each degree, hub residues of NCS1 and their conservation in UniRef50 cluster are reported.

Deg	Rec-R					Rec-GRK1				
**8**	D74 (100)	A89 (100)	D110 (100)			D74 (100)	D110 (100)			
**7**	F49 (99.2)	V87 (37.7)	I88 (84.4)	A105 (100)	M132 (65.6)	F57 (99.2)	K101 (100)	M132 (65.6)	E145 (99.2)	A152 (81.1)
	I155 (45.1)					I155 (45.1)	E181 (46.7)	K194 (45.1)		
**6**	E12 (96.7)	E16 (39.3)	R71 (100)	E85 (100)	M92 (75.4)	E12 (96.7)	I13 (84.4)	L14 (99.2)	F35 (100)	I52 (99.2)
	W104 (100)	E121 (99.2)	R151 (98.4)	F172 (92.6)		L90 (100)	W104 (100)	A105 (100)	F106 (100)	L108 (98.4)
						E121 (99.2)	V122 (92.6)	I129 (99.2)	K154 (98.4)	
**5**	S10 (94.3)	I13 (84.4)	L28 (68.9)	W31 (99.2)	Y53 (99.2)	E16 (39.3)	L28 (68.9)	W31 (99.2)	C39 (96.7)	F49 (99.2)
	F57 (99.2)	Q67 (64.8)	H68 (100)	V69 (100)	F70 (100)	Y53 (99.2)	F56 (99.2)	Q67 (64.8)	H68 (100)	F70 (100)
	F73 (100)	D78 (100)	D82 (100)	L90 (100)	F106 (100)	R71 (100)	E85 (100)	H91 (96.7)	S107 (89.3)	I125 (95.9)
	L108 (98.4)	V122 (92.6)	A128 (82)	T147 (95.9)	A152 (81.1)	A128 (82)	T147 (95.9)	R151 (98.4)	W156 (96.7)	F172 (92.6)
	K154 (98.4)	W156 (96.7)	F159 (95.9)	D164 (44.2)	N179 (87.7)	N179 (87.7)	I182 (57.4)	R184 (87.7)	I186 (91.8)	P190 (49.2)
	I182 (57.4)	L183 (88.5)	L185 (92.6)			V193 (42.6)	E195 (39.3)	K196 (28.7)	L197 (84.4)	
**4**	K11 (91)	F35 (100)	C39 (96.7)	R43 (91)	Q50 (35.2)	L9 (64.8)	S10 (94.3)	K11 (91)	N20 (74.6)	K22 (77.9)
	T51 (8.2)	I52 (99.2)	P62 (99.2)	L81 (98.4)	F83 (100)	S34 (54.9)	R43 (91)	E48 (77)	Q50 (35.2)	T51 (8.2)
	K84 (86.9)	S107 (89.3)	D112 (100)	T116 (29.5)	K119 (99.2)	P62 (99.2)	V69 (100)	D78 (100)	D82 (100)	F83 (100)
	E124 (97.5)	I125 (95.9)	V126 (41.8)	I133 (84.4)	S134 (18)	K84 (86.9)	Y86 (96.7)	V87 (37.7)	I88 (84.4)	A89 (100)
	T138 (1.6)	E145 (99.2)	K150 (97.5)	E153 (43.4)	F158 (54.1)	M92 (75.4)	S94 (77.9)	N99 (25.4)	Q100 (45.1)	L102 (100)
	K161 (96.7)	E169 (91)	I173 (88.5)	G175 (88.5)	R184 (87.7)	Y109 (51.6)	D112 (100)	K119 (99.2)	E124 (97.5)	V126 (41.8)
	I186 (91.8)					F130 (98.4)	I133 (84.4)	S134 (18)	D137 (49.2)	T138 (1.6)
						P142 (91)	D144 (98.4)	N146 (94.3)	K150 (97.5)	E153 (43.4)
						F159 (95.9)	K161 (96.7)	I173 (88.5)	G175 (88.5)	L183 (88.5)
						Q187 (84.4)	F188 (41)	E189 (45.1)	K192 (52.5)	E199 (45.1)

Table ST3. Hubs for PSN of Rec-R and Rec-GRK1 states. For each degree, hub residues of NCS1 and their conservation in UniRef50 cluster are reported.

Rec - GRK1					NCS1 - D_2R					NCS1 - GRK1				
F23[a]	W31[a]	F35[a]	F49[a]	I52[a]	W30[c]	F34[c]	F48[c]	I51[c]	Y52[c]	W30[c]	I51[c]	Y52[c]	F55[c]	F64[c]
Y53[a]	F56[a]	F57[a]	Y86[a]	L90[a]	F55[c]	F56[c]	V68[c]	F72[c]	F85[c]	V68[c]	F85[c]	L89[c]	L101[c]	W103[c]
E189[b]	P190[b]	Q191[b]	K192[b]	V193[b]	L89[c]	W103[c]	L107[c]	Y108[c]	I128[c]	A104[c]	L107[c]	Y108[c]	M121[c]	I124[c]
					Y129[c]	Q130[c]	M131[c]	V132[c]	V136[c]	V125[c]	I128[c]	M131[c]	I152[c]	M156[c]
					L138[c]	I152[c]	M156[c]	F169[c]	I179[c]	F169[c]	I179[c]	A182[c]	L183[c]	
					V180[c]	A182[c]	L183[c]							

Table ST4. List of interface residues of Rec and NCS1 with their targets. Interface residues were identified in: [a] (Ames et al., 2006), [b] (Zernii et al., 2011), [c] (Pandalaneni et al., 2015).

Deg	Ca^{2+}-loaded GCAP1					EF2-Mg^{2+} GCAP1				
8	D64 (99)	D100 (97)				W21 (99)	D64 (99)			
7	W21 (99)	R40 (72.3)	F96 (97)	D144 (97)	V160 (94.1)	R40 (72.3)	A78 (99)	F96 (97)	D100 (97)	D175 (96)
6	A52 (88.1)	F60 (99)	D72 (99)	E111 (96)	I119 (97)	A52 (88.1)	F60 (99)	I116 (97)	I119 (97)	F135 (97)
	I143 (97)	D163 (97)	L166 (95)	D175 (96)		V139 (98)	D144 (97)	V160 (94.1)	D163 (97)	L176 (96)
5	V10 (93.1)	F25 (99)	L45 (96)	V56 (94.1)	M59 (99)	V10 (93.1)	F25 (99)	V56 (94.1)	M59 (99)	E61 (92.1)
	F73 (99)	E75 (99)	A78 (99)	A79 (99)	K91 (97)	D72 (99)	F73 (99)	E75 (99)	A79 (99)	L84 (99)
	L92 (97)	Y95 (97)	I122 (97)	V139 (98)	L170 (95)	K91 (97)	L92 (97)	R93 (95)	Y95 (97)	R109 (95)
	L176 (96)	V180 (95)				I122 (97)	S126 (68.3)	F156 (97)	L166 (95)	T171 (95)
4	L13 (93.1)	C29 (99)	F43 (99)	S53 (69.3)	Y55 (99)	E11 (91.1)	L13 (93.1)	E17 (99)	Y22 (99)	C29 (99)
	E57 (99)	D68 (99)	L82 (99)	L84 (99)	R93 (95)	L36 (95)	F39 (99)	F43 (99)	L45 (96)	S53 (69.3)
	W94 (97)	Y99 (97)	D108 (96)	R109 (95)	T114 (67.3)	Y55 (99)	E57 (99)	F63 (99)	F65 (96)	D68 (99)
	I115 (96)	I116 (97)	R120 (98)	A121 (80.2)	S126 (68.3)	L80 (99)	L82 (99)	V83 (99)	W94 (97)	T114 (67.3)
	F135 (97)	F140 (97)	D148 (97)	E155 (97)	F156 (97)	A121 (80.2)	A132 (95)	F140 (97)	L153 (97)	I157 (43.5)
	I157 (43.5)	D168 (86.1)	T171 (95)	I179 (96)	R182 (66.3)	D168 (86.1)	L170 (95)	R172 (93.1)	R178 (56.4)	R181 (79.2)
	L183 (65.3)					L183 (65.3)				

Table ST5. Hubs for PSN of Ca^{2+}-loaded and EF2-Mg^{2+} GCAP1 states. For each degree, hub residues of GCAP1 and their conservation in UniRef50 cluster are reported. Data are from simulations reported in (Marino and Dell'Orco, 2016)

Supplementary references

Ames, J.B., Hamasaki, N., and Molchanova, T. (2002). Structure and calcium-binding studies of a recoverin mutant (E85Q) in an allosteric intermediate state. *Biochemistry* 41**,** 5776-5787.

Ames, J.B., Ishima, R., Tanaka, T., Gordon, J.I., Stryer, L., and Ikura, M. (1997). Molecular mechanics of calcium-myristoyl switches. *Nature* 389**,** 198-202.

Ames, J.B., Levay, K., Wingard, J.N., Lusin, J.D., and Slepak, V.Z. (2006). Structural basis for calcium-induced inhibition of rhodopsin kinase by recoverin. *J Biol Chem* 281**,** 37237-37245.

Hess, B. (2002). Convergence of sampling in protein simulations. *Phys Rev E Stat Nonlin Soft Matter Phys* 65**,** 031910.

Marino, V., and Dell'Orco, D. (2016). Allosteric communication pathways routed by Ca(2+)/Mg(2+) exchange in GCAP1 selectively switch target regulation modes. *Sci Rep* 6**,** 34277.

Pandalaneni, S., Karuppiah, V., Saleem, M., Haynes, L.P., Burgoyne, R.D., Mayans, O., Derrick, J.P., and Lian, L.Y. (2015). Neuronal Calcium Sensor-1 Binds the D2 Dopamine Receptor and G-protein-coupled Receptor Kinase 1 (GRK1) Peptides Using Different Modes of Interactions. *J Biol Chem* 290**,** 18744-18756.

Tanaka, T., Ames, J.B., Harvey, T.S., Stryer, L., and Ikura, M. (1995). Sequestration of the membrane-targeting myristoyl group of recoverin in the calcium-free state. *Nature* 376**,** 444-447.

Weiergraber, O.H., Senin, Ii, Philippov, P.P., Granzin, J., and Koch, K.W. (2003). Impact of N-terminal myristoylation on the Ca2+-dependent conformational transition in recoverin. *J Biol Chem* 278**,** 22972-22979.

Zernii, E.Y., Komolov, K.E., Permyakov, S.E., Kolpakova, T., Dell'Orco, D., Poetzsch, A., Knyazeva, E.L., Grigoriev, Ii, Permyakov, E.A., Senin, Ii, Philippov, P.P., and Koch, K.W. (2011). Involvement of the recoverin C-terminal segment in recognition of the target enzyme rhodopsin kinase. *Biochem J* 435**,** 441-450.
